# Optimised MobileNet for very lightweight and accurate plant leaf disease detection

**DOI:** 10.1038/s41598-025-27393-z

**Published:** 2025-12-12

**Authors:** Vincent Nnamdi Ugwah, Vahid Abolghasemi

**Affiliations:** 1 School of Computer Science and Electronics Engineering, University of Essex, Colchester, UK; 2 School of Engineering and Engineering Technology, Federal University of Technology Owerri, Owerri, Imo state Nigeria

**Keywords:** Plant leaf Disease Detection, Deep Learning, MobileNet, Multi-Class Classification, Classification and taxonomy, Fungal infection, Plant ecology, Machine learning

## Abstract

The development of accurate and efficient plant disease classification systems is vital for addressing the challenges of climate change and the growing global demand for food. This study presents $\hbox {V}^2$PlantNet, a novel lightweight multi-class classification model based on a modified MobileNet architecture, designed to detect plant leaf diseases across a diverse range of crop types. $\hbox {V}^2$PlantNet employs depthwise separable convolutions to significantly reduce model complexity without compromising accuracy. The architecture integrates Batch Normalization (BN) and Rectified Linear Unit (ReLU) activation after each convolutional layer, while a multi-stage design enhances feature extraction and overall performance. Despite its compact size, comprising only 389,286 parameters and requiring just 1.46 MB of memory, $\hbox {V}^2$PlantNet achieved up to 99% training accuracy, with validation and test accuracies of 97% and 98%, respectively. Across most classes, precision, recall, and F1-scores ranged from 0.97 to 1.0, demonstrating consistent and robust generalization across diverse plant species. These architectural innovations enable $\hbox {V}^2$PlantNet to outperform larger models such as ResNet-50 and Inception V3 in terms of computational efficiency, owing to its smaller model size (1.46 MB), reduced parameter count (389,286), and faster inference time (0.676 s), offering a scalable solution for real-time plant disease detection in precision agriculture.

## Introduction

Agriculture is fundamental to global food security, employment, and economic development, especially in underdeveloped areas^1^. According to the Food and Agriculture Organization (FAO), it accounted for 26% of the global workforce in 2024^2^, contributing approximately 4% of the global gross domestic product (GDP) and exceeding 25% in low-income economies, underscoring its role in poverty reduction and economic stability. However, given that the population is projected to grow to 9.2 billion people by 2050, studies indicate that global food production must increase substantially by an estimated 60–70% or even double to meet the growing demand^3,4^. Despite global efforts to alleviate hunger, the agricultural sector continues to face significant challenges due to climate change and a rapidly expanding global population.

Among the most immediate threats to crop productivity are plant leaf diseases, which reduce yields and compromise food quality. Globally, pests and diseases account for substantial crop yield losses, with estimates averaging 21.5% for wheat, 30% for rice, 22. 5% for maize, 17. 2% for potato and 21. 4% for soybean^5^. These losses in staple crops such as rice, wheat, maize and potatoes directly threaten food security and nutrition, resulting in significant economic consequences at the household and national levels^6^. Addressing these challenges and minimizing crop losses is therefore essential for enhancing productivity and ensuring resilient food systems. As demand for food and feed crops increases, there is an urgent need for quick, non-invasive methods for the evaluation of field crop health^7^. Therefore, the importance of efficient screening and selection of plant traits that contribute to higher yields under varying genetic, environmental and management practices cannot be overemphasized.

Diseases affecting plant leaves, such as canker, black rot, early blight, and rust, substantially affect plant vitality^8–10^. These diseases often lead to nutritional deficiencies, poor leaf quality, and reduced plant productivity, thus exacerbating global food security issues^9–11^. Recent advances in image-sensing technologies have highlighted the potential of automating plant disease detection. However, accurate detection remains a challenge due to the small size of lesions and the low contrast between diseased and healthy tissue^12^. For example, visual symptoms such as yellow patches with dark spots caused by Alternaria blight in jasmine leaves can progress to larger lesions with concentric rings, complicating detection^11^.

Traditionally, detecting plant leaf diseases has relied on human experts or farmers visually inspecting crops. This method is slow, error-prone, and often misleading^9^. However, recent advances in computer vision, incorporating machine learning, deep learning, and image processing techniques, have significantly improved the detection of plant diseases^13^. For example, Tian et al.^14^ employed Support Vector Machines (SVMs) to classify wheat diseases such as powdery mildew and rust. In contrast, Rumpf et al.^15^ used hyperspectral reflectance data and SVMs for early detection of plant leaf diseases. Other studies, such as Sheelavantamath et al.^16^, integrated image segmentation with clustering and Otsu’s method for enhanced disease detection. Elangovan et al.^17^ further explored the combination of segmentation techniques with SVMs for accurate disease classification. Another study by Majumdar et al.^18^ utilized fuzzy c-means clustering combined with an Artificial Neural Network (ANN) to classify disease features in wheat. One key limitation of many machine learning models is their struggle to handle large datasets with complex lesion features across different leaf regions^19^. In addition, machine learning models typically require manual feature extraction, where experts must identify and describe specific features for disease detection, a process that is both labour-intensive and knowledge-intensive^20^.

Convolutional Neural Networks (CNNs) have emerged as a promising solution to address these challenges. CNNs process image data efficiently by eliminating the need for manual feature extraction, making them more advantageous than traditional machine learning techniques^20,21^. Furthermore, CNNs significantly improve the accuracy of plant disease identification by transforming image features into new feature spaces through layered transformations, enhancing their performance compared to conventional machine learning models^22^.

Recent studies have investigated the application of CNNs for automated disease identification in crops. Ferentinos et al.^23^ evaluated CNN models for identifying 58 diseases in 25 plant species from the PlantVillage dataset, while Liang et al.^24^ developed the PD2SE-Net model to detect crop diseases and evaluate their severity. Bao et al.^25^ proposed an elliptical-maximum margin criterion algorithm for identifying wheat leaf diseases and measuring their severity. Other studies have focused on specific crop diseases^25^, such as Jin et al.^26^, who reconstructed spectral data using CNNs to classify wheat ear health, and Su et al.^27^, who incorporated a local SVM classifier into CNNs for improved handling of imbalanced datasets in wheat disease detection. However, many of these models face limitations when applied to diverse crop datasets due to their crop-specific designs^19^. Their computational complexity and large number of parameters also make them unsuitable for deployment in low-power devices or in real-time settings. In rural areas with limited access to high-performance computing, these models are often impractical. Additionally, when applied to small datasets, complex models tend to overfit, learning the training data too well and failing to generalize effectively to new data. Models with a large number of parameters may also underfit, resulting in poor performance on training data. Despite attempts to optimize computational efficiency, many models still lack focus on reducing model size and parameter count while maintaining high detection accuracy across various crops^28^.

To address these challenges, lightweight models balancing high accuracy with computational efficiency are urgently needed. Such models would facilitate real-time, in-field disease detection on low-power devices, enabling timely interventions for disease management. The central questions of this research are: 1) “How effective is a MobileNet-based architecture for multi-class classification of plant diseases across diverse crops in terms of accuracy and computational efficiency?” and 2) “How well does it generalize across different crops?”. This study develops and optimizes a lightweight model, $\hbox {V}^2$PlantNet (short for Vincent-Vahid-PlantNet), built on a modified MobileNet architecture, to answer this question. The primary contributions of this research are as follows:

1. Development of a novel lightweight model: We introduce $\hbox {V}^2$PlantNet, a multi-class classification model based on a modified MobileNet architecture, designed to efficiently and accurately classify plant-leaf diseases, with a focus on both performance and computational efficiency.
2. Comprehensive training on a diverse dataset: $\hbox {V}^2$PlantNet was trained on a dataset containing 38 labelled classes from 14 crop types. Data augmentation techniques were applied solely to the training data generator, while validation and test sets consisted of original images to ensure unbiased evaluation.
3. Rigorous model evaluation and benchmarking: $\hbox {V}^2$PlantNet was evaluated using standard metrics such as accuracy, precision, recall, sensitivity, specificity, F1-score, and Matthews Correlation Coefficient (MCC). Additionally, the model’s computational efficiency was assessed through factors like floating-point operations (FLOPs), memory usage, model size, and inference time. $\hbox {V}^2$PlantNet’s performance was compared against other MobileNet variants and state-of-the-art models, including AlexNet, EfficientNet, DenseNet-121, XceptionNet, ResNet-50, and SoyNet.
4. Enhanced computational efficiency and deployment suitability: $\hbox {V}^2$PlantNet demonstrates lower computational complexity and greater cost-effectiveness while maintaining or surpassing the accuracy of existing models. This makes it particularly well-suited for deployment in resource-limited settings and real-time applications where computational efficiency is essential.

The remainder of the paper is organized as follows. Section Related work reviews the related work. Section Methodology presents the proposed methodology in detail. Section Experimental setup and procedure describes the experimental setup and procedure. Section Results reports and discusses the results, including a comprehensive comparison with state-of-the-art methods. Finally, Section Conclusion concludes the paper.

## Related work

MobileNet has emerged as a prominent architecture in plant disease detection and classification research, particularly where achieving a balance between accuracy and computational efficiency is critical. Its lightweight design and capacity to perform well in resource-constrained environments have generated substantial attention in agricultural applications.

Several studies have explored the efficacy of the MobileNet model in plant disease classification. For instance, Kamal et al.^29^ integrated a deep separable convolutional architecture within a MobileNet framework, showcasing its potential for precise disease classification. Their study achieved an impressive 98.34% classification accuracy on the widely used PlantVillage dataset, affirming the model’s capability in plant disease detection. Furthermore, the study evaluated different architectures, noting that the Reduced MobileNet model had 29 times fewer parameters compared to the VGG architecture and six times fewer than the standard MobileNet, making it highly suitable for deployment in environments with limited computational resources. This reduced parameter count, coupled with a 36.03% accuracy on a separate test set, highlights the essential balance MobileNet offers between efficiency and accuracy, positioning it as an ideal choice for real-time crop diagnostics on mobile devices.

In another study, Ashwinkumar et al.^30^ proposed an Optimized Mobile Network-based Convolutional Neural Network (OMNCNN) designed specifically for automated plant leaf disease detection. Their approach involved preprocessing with bilateral filtering and segmentation using Kapur’s thresholding to isolate affected areas on plant leaves. The MobileNet architecture was employed for feature extraction, followed by hyperparameter tuning using the Emperor Penguin Optimizer (EPO) to improve classification performance. The model utilized an extreme learning machine (ELM) classifier for labelling, and simulation analyses demonstrated the model’s effectiveness in disease detection, further validating MobileNet’s potential for use in precision agriculture.

In a similar study, Gavai et al.^31^ investigated the application of MobileNet in flower classification, underscoring its efficiency in reducing both time and space requirements without significantly compromising accuracy. When trained on the Oxford-102 flower dataset, MobileNet achieved classification accuracies between 85% and 99%, with higher precision observed when fewer categories were considered. The study acknowledged the inherent challenges in flower classification due to species similarities, yet concluded that MobileNet outperformed more complex models such as Google’s Inception-v3 in terms of resource efficiency. The inclusion of width and resolution multipliers further minimized the model size and latency, reinforcing the practicality of MobileNet for real-world applications.

Senthil et al. propose the Gradient-Weighted DenseNet-201 (GradWDN-201) model, combining DenseNet-201 with Gradient Weighted Class Activation Mapping and texture features (GLCM), achieving high accuracies across multiple benchmark datasets^32^. Another study presents a Hybrid Crossover Oppositional Firefly Optimization framework applied to transfer learning for leaf disease classification, improving parameter search and classification robustness^33^. A separate work integrates a Wild Horse Optimizer with a convolutional-attention BiLSTM architecture to refine feature extraction and temporal/contextual modelling for disease detection^34^. Further, an approach deploys enhanced deep-learning pipelines, leveraging refined preprocessing and ensemble strategies to increase diagnostic robustness against dataset variation^35^. These contributions demonstrate strong gains in classification performance, but largely focus on curated datasets and optimization of model accuracy; they underexplore architectural efficiency, and cross-domain generalization.

Sharma et al.^12^ introduced the DLMC-Net model, designed to address issues like the vanishing gradient problem by incorporating collective blocks and a passage layer. The model leverages point-wise and separable convolution blocks, achieving accuracy rates between 93.56% and 99.50% across four crop types: citrus, cucumber, grapes, and tomato. With a low parameter count of 6.4 million, DLMC-Net represents a lightweight solution for plant-leaf disease classification. The model outperformed seven state-of-the-art approaches across metrics like precision, recall, and F1-score, further demonstrating its robustness for real-time agricultural applications. The reduced computational demand of DLMC-Net enhances its utility in resource-limited settings, allowing for timely interventions and improved crop management practices.

Furthermore, Bao et al.^22^ introduced SimpleNet, a lightweight CNN specifically designed for the automatic identification of wheat ear diseases. Built upon the MobileNet architecture, and to improve feature extraction and representation, SimpleNet uses convolutional and inverted residual blocks, as well as a Convolutional Block Attention Module (CBAM). Despite having only 2.13 million parameters, SimpleNet scored an impressive 94.1% accuracy, outperforming standard CNN models like VGG16, ResNet50, and AlexNet, as well as lightweight designs like MobileNet Version 1,2 and 3. SimpleNet’s efficient design and high accuracy make it an excellent choice for mobile agricultural applications, giving a strong tool for disease identification in real time on the field.

While CNN-based architectures such as MobileNet prioritize lightweight efficiency, recent studies have increasingly explored the potential of transformer models for plant disease detection. Murugavalli and Gopi^36^ developed PLA-ViT, a Vision Transformer (ViT)-based model specifically designed for precision agriculture applications. Unlike conventional CNNs that operate on localized receptive fields, PLA-ViT employs self-attention mechanisms to capture long-range dependencies and global contextual relationships within leaf images. Using transfer learning from large-scale ViT models and image enhancement techniques such as bilateral filtering, PLA-ViT achieved an accuracy of 98.7% on the evaluated dataset, surpassing multiple CNN-based baselines.

Similarly, Karthik et al.^37^ proposed a hybrid CNN-Transformer framework that combines a Swin Transformer with a Dual-Attention Multiscale Fusion Network (DAMFN) to capture global dependencies and localized disease patterns. The architecture incorporates multiscale attention blocks and a triplet attention mechanism, enabling selective focus on discriminative image regions. When evaluated on the CCMT dataset, the hybrid model achieved an accuracy of 95.68%, outperforming several state-of-the-art CNN and transformer architectures. These findings underscore the effectiveness of hybrid CNN–Transformer approaches in mitigating the limitations of pure CNNs and transformer-only models by enhancing robustness to scale variations and reducing false positive.

Building upon these advances, the proposed $\hbox {V}^2$PlantNet model aims to balance the computational efficiency of MobileNet with the representational capacity of transformer-based architectures such as PLA-ViT and hybrid CNN–Transformer networks. Although MobileNet variants demonstrate strong performance in resource-constrained environments, they exhibit limited ability to capture complex spatial dependencies associated with subtle disease manifestations. In contrast, transformer-based models provide superior global feature modeling but incur high computational costs and data requirements, restricting their use in practical field deployments. To address this trade-off, $\hbox {V}^2$PlantNet introduces an optimized MobileNet variant that preserves architectural compactness while improving feature extraction and generalization. By reducing parameter count and memory utilization without compromising accuracy, the model provides a scalable and efficient solution for the real-time detection of plant disease in low-resource agricultural settings.

## Methodology

**Fig. 1:**
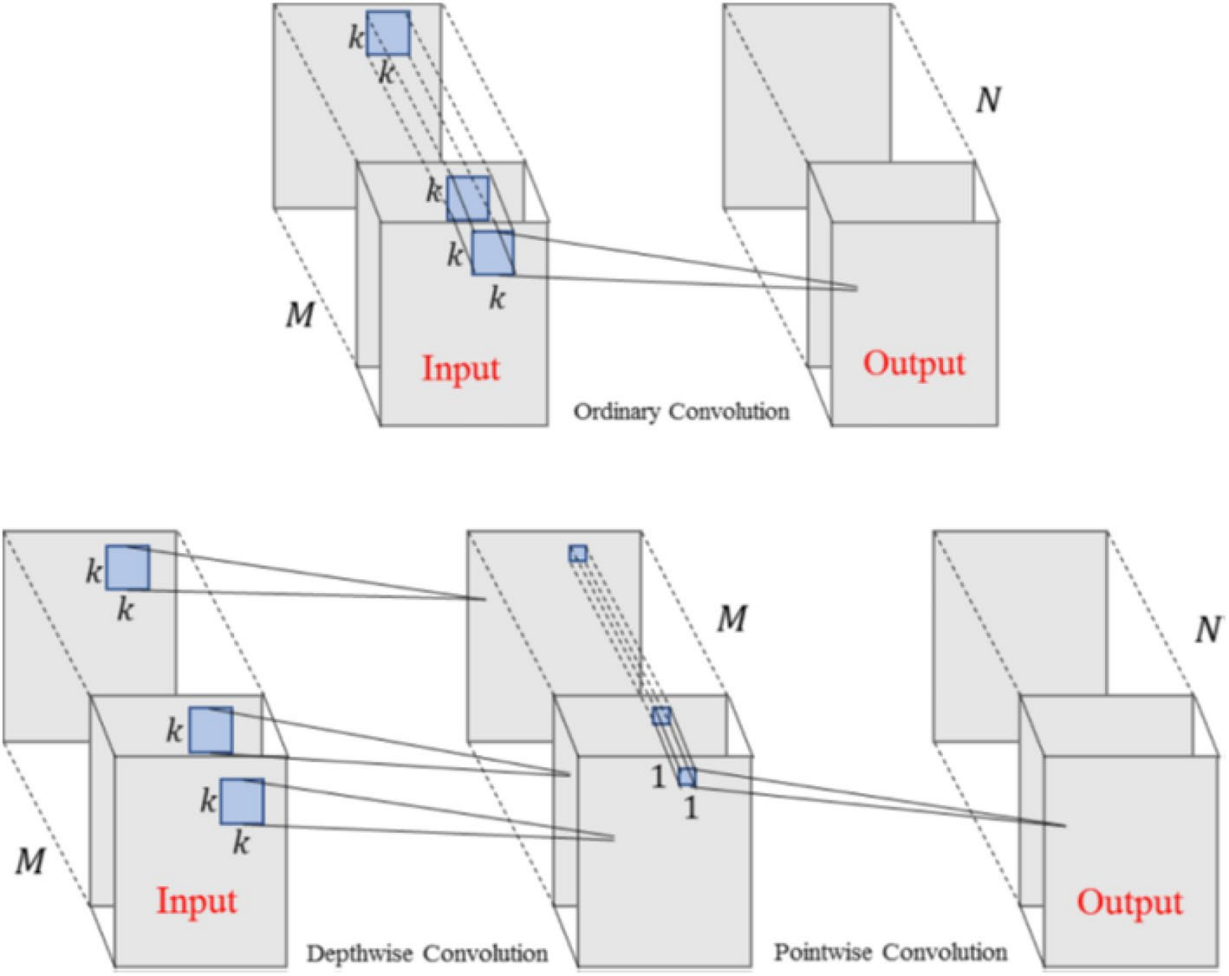
Schematic of ordinary convolution and depthwise separable convolution^22^.

The development of the proposed model is based on the MobileNet V1 architecture, adapted specifically for image classification tasks on the PlantNet dataset. This section outlines the architecture of the model’s unique features and the methodologies used for its development and optimization. The MobileNet V1 architecture uses separable depth convolutions, which break down the traditional convolution operation into two distinct processes: depth convolution and point convolution, as depicted in Fig. 1.

### Convolutional operations comparison

In the depthwise convolution, a separate convolutional filter is independently applied to each input channel. This operation is expressed as:

1 $$\begin{aligned} {\textbf{Y}}_{dw}(i, j, c) = \sum _{m=0}^{M-1} \sum _{n=0}^{N-1} {\textbf{X}}(i+m, j+n, c) \cdot {\textbf{K}}_{dw}(m, n, c), \end{aligned}$$

where $\textbf{K}_{dw}$ is the Depthwise Convolution kernel of size $M \times N \times 1$, and the output $\textbf{Y}_{dw}$ has dimensions $H' \times W' \times C$. Following this layer, a pointwise convolution (a $1 \times 1$ convolution) combines the independently processed channels:

2 $$\begin{aligned} \textbf{Y}_{pw}(i, j, k) = \sum _{c=0}^{C-1} \textbf{Y}_{dw}(i, j, c) \cdot \textbf{K}_{pw}(c, k) + \textbf{B}_{pw}(k), \end{aligned}$$

where $\textbf{K}_{pw}$ is the pointwise convolution kernel with dimensions $1 \times 1 \times C \times K$, and the output $\textbf{Y}_{pw}$ has dimensions $H' \times W' \times K$, combining features from all input channels into $K$ output channels. Table 1 shows the detailed definitions of symbols used in this study.

Traditional convolutions require $M \times N \times C \times K$ parameters, while depthwise and pointwise separable convolutions require $M \times N \times C$ and $1 \times 1 \times C \times K$, respectively, which reduces computational complexity^12^.

### Proposed architecture

The proposed model improves upon the original MobileNet V1 architecture by integrating Batch Normalization (BN) and ReLU activation after each convolutional layer, addressing several key limitations. MobileNet V1, though efficient, suffers from training instability, slower convergence, and reduced performance in deeper layers. These issues are largely due to the absence of BN and ReLU, resulting in internal covariate shifts and limited non-linearity. By incorporating BN and ReLU after both depthwise and pointwise convolution layers, the proposed model stabilizes the training process, reduces covariate shift, and enables faster convergence. ReLU further introduces the non-linearity required for learning more complex patterns, all while maintaining computational efficiency.

Additionally, the model retains the core structure of MobileNet with repeated MobileNet blocks, starting with depthwise convolution followed by BN and ReLU, and concluding with pointwise convolution, again followed by BN and ReLU. This design improves gradient flow, minimizing the risk of vanishing gradients and enhancing the model’s capacity to learn deeper features. The first block in each stage employs downsampling to reduce spatial dimensions, while subsequent blocks preserve them with a stride of 1, maintaining the model’s lightweight nature. These modifications not only resolve the limitations of MobileNet V1 but also ensure that the model remains efficient and suitable for mobile and embedded applications. The proposed modified process is as follows. For depthwise convolution:

3 $$\begin{aligned} \textbf{Y}_{dw}(i, j, c) = \sum _{m=0}^{M-1} \sum _{n=0}^{N-1} \textbf{X}(i+m, j+n, c) \cdot \textbf{K}_{dw}(m, n, c), \end{aligned}$$

followed by BN:

4 $$\begin{aligned} \hat{\textbf{Y}}_{dw}(i, j, c) = \text {BN}(\textbf{Y}_{dw}(i, j, c)), \end{aligned}$$

and ReLU Activation:

5 $$\begin{aligned} \textbf{Y}^{\text {ReLU}}_{dw}(i, j, c) = \max (0, \hat{\textbf{Y}}_{dw}(i, j, c)). \end{aligned}$$

For pointwise convolution:

6 $$\begin{aligned} \textbf{Y}_{pw}(i, j, k) = \sum _{c=0}^{C-1} \textbf{Y}^{\text {ReLU}}_{dw}(i, j, c) \cdot \textbf{K}_{pw}(c, k) + \textbf{B}_{pw}(k), \end{aligned}$$

followed by BN:

7 $$\begin{aligned} \hat{\textbf{Y}}_{pw}(i, j, k) = \text {BN}(\textbf{Y}_{pw}(i, j, k)), \end{aligned}$$

and ReLU Activation:

8 $$\begin{aligned} \textbf{Y}^{\text {ReLU}}_{pw}(i, j, k) = \max (0, \hat{\textbf{Y}}_{pw}(i, j, k)). \end{aligned}$$

**Table 1:** Notation definitions used in the equations.

Symbol	Description
$\textbf{Y}_{dw}(i, j, c)$	Output of Depthwise Convolution at spatial position (*i*, *j*) for channel *c*.
$\textbf{X}(i+m, j+n, c)$	Input feature map at spatial position $(i+m, j+n)$ for channel *c*.
$\textbf{K}_{dw}(m, n, c)$	Depthwise convolution kernel for channel *c*, applied over a region of size $M \times N \times 1$.
*M*, *N*	Spatial dimensions of the Depthwise Convolution kernel.
$H', W'$	Height and width of the output feature map after the Depthwise Convolution.
*C*	Number of input channels in the Depthwise Convolution.
$\hat{\textbf{Y}}_{dw}(i, j, c)$	BN output of Depthwise Convolution at spatial position (*i*, *j*) for channel *c*.
$\textbf{Y}^{\text {ReLU}}_{dw}(i, j, c)$	ReLU-activated output of Depthwise Convolution for channel *c*.
$\textbf{Y}_{pw}(i, j, k)$	Output of Pointwise Convolution at position (*i*, *j*) for output channel *k*.
$\textbf{K}_{pw}(c, k)$	Pointwise convolution kernel between input channel *c* and output channel *k*, with dimensions $1 \times 1 \times C \times K$.
$\textbf{B}_{pw}(k)$	Bias term for output channel *k*.
*K*	Number of output channels in the Pointwise Convolution.
$\hat{\textbf{Y}}_{pw}(i, j, k)$	BN output of Pointwise Convolution for output channel *k*.
$\textbf{Y}^{\text {ReLU}}_{pw}(i, j, k)$	ReLU-activated output of Pointwise Convolution for output channel *k*.
$\text {BN}(\cdot )$	BN operation applied to the input.
$\max (0, \cdot )$	ReLU activation function.

More so, this modified architecture maintains the computational efficiency of depthwise separable convolutions while benefiting from the regularization provided by BN and the flexibility of ReLU activation. These improvements ensure that the model performs efficiently and exhibits greater robustness during training.

As outlined in Table 2, the input dimensions are set at (224, 224, 3), indicating that the model processes 224x224 RGB images. The architecture commences with an initial convolutional layer that applies 32 filters with a 3x3 kernel and a stride of 2 to downsample the input. This initial layer is followed by BN and a ReLU activation function, promoting stability and non-linearity within the model. Subsequently, a MaxPooling layer with a 3x3 pool size and a stride of 2 is employed to further downsample the spatial dimensions.

The primary structure of the model is organized into several stages of MobileNet blocks. Each block begins with a depthwise convolution layer using a 3x3 kernel and predefined strides, followed by BN and ReLU activation. A subsequent pointwise convolution layer (1x1 kernel) also includes BN and ReLU activation, effectively separating the feature extraction into two stages. The integration of BN and ReLU activation between depthwise and pointwise convolution layers optimizes training efficiency and stability, as BN stabilizes the learning process and ReLU introduces non-linearity, enabling the model to capture complex patterns. Repeating these blocks within each stage enhances the model’s efficiency and speed, which is advantageous for lightweight architectures like the one proposed. In each stage, the first block is configured for downsampling (using strides greater than 1), while subsequent blocks maintain the spatial dimensions by using a stride of 1.

**Fig. 2:**
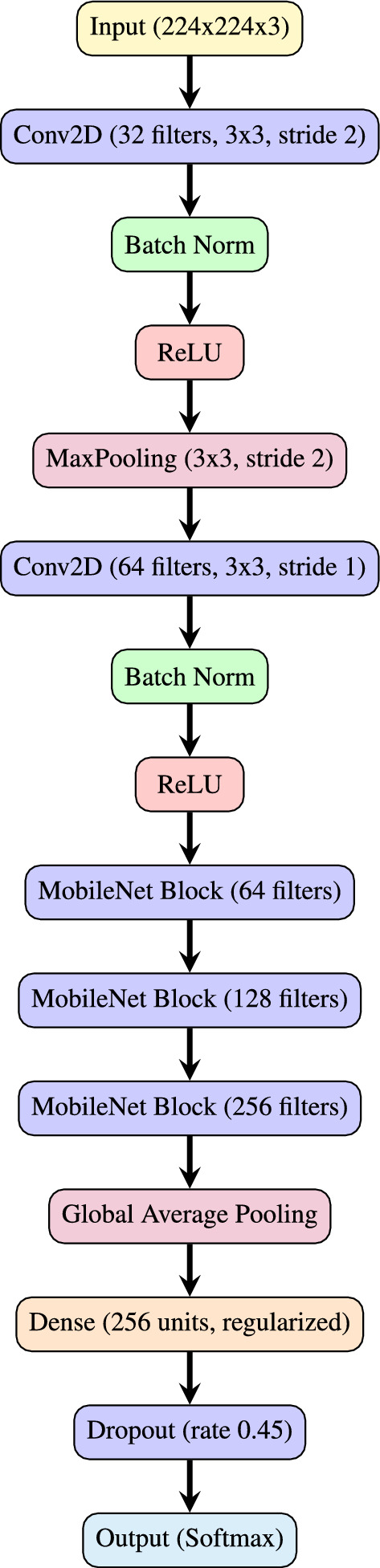
Architectural flow path of the proposed $\hbox {V}^2$PlantNet model.

The proposed architecture, as depicted in Fig. 2, comprises three main stages, each with distinct repetitions, filter counts, and strides. The first stage utilizes 64 filters, repeated 3 times, the second stage uses 128 filters, repeated 7 times, and the third stage applies 256 filters, repeated 3 times. In the first stage, three repetitions of depthwise separable convolution blocks are employed, each containing 64 filters and using a stride of 1. This configuration preserves the spatial resolution of the input tensor while expanding the depth to 64 channels, facilitating initial feature extraction without altering spatial dimensions. The increased filter count at this stage enables the model to capture richer feature representations.

In the second stage, the model includes seven repetitions with 128 filters, starting with a stride of 2 for the initial block. This stride halves the spatial dimensions, decreasing computational costs and emphasizing more abstract feature learning. The increased depth to 128 channels allows the model to discern more detailed patterns. The subsequent blocks within this stage retain a stride of 1, maintaining the reduced spatial resolution and refining the extracted features essential for mid-level representation.

The third stage intensifies these effects by employing three repetitions with 256 filters, where the first block begins with a stride of 2, further reducing spatial dimensions and concentrating the network’s capacity on higher-level features. With a depth of 256 channels, this stage enables the model to capture intricate and abstract patterns, which are critical for high-level feature extraction and accurate classification.

This design strategically balances model complexity with computational efficiency. Following the convolutional stages, a global average pooling layer reduces spatial dimensions to a single vector, encapsulating essential features from previous layers. A dense layer with 256 units follows, incorporating L2, L1 activity, and L1 bias regularizers to mitigate overfitting. Additionally, a dropout layer with a rate of 0.45 enhances regularization. The architecture concludes with a dense layer, containing units equal to the number of classes and employing a softmax activation function to output class probabilities. This streamlined design supports compactness and computational efficiency, rendering it suitable for efficient image classification tasks.

**Table 2:** $\hbox {V}^2$PlantNet model architecture and output shapes.

Stage	Layer Type	Output Configurations	Repeat
Initial	Input	(None, 224, 224, 3) Input shape	1
1	Conv2D	(None, 112, 112, 32) 3x3 conv, stride-2, BN, ReLU	1
1	Max Pooling	(None, 56, 56, 32) 3x3 pool, stride-2	1
1	Depthwise Conv2D	(None, 56, 56, 32) 3x3 conv, BN, ReLU	1
1	Conv2D	(None, 56, 56, 64) 1x1 conv, BN, ReLU	1
2	Depthwise Conv2D	(None, 56, 56, 64) 3x3 conv, BN, ReLU	2
2	Conv2D	(None, 28, 28, 128) 1x1 conv, BN, ReLU, stride-2	1
2	Depthwise Conv2D	(None, 28, 28, 128) 3x3 conv, BN, ReLU	5
3	Conv2D	(None, 14, 14, 256) 1x1 conv, BN, ReLU, stride-2	1
3	Depthwise Conv2D	(None, 14, 14, 256) 3x3 conv, BN, ReLU	2
3	Conv2D	(None, 14, 14, 256) 1x1 conv, BN, ReLU	1
Final	Global Average Pooling	(None, 256)	1
Final	Fully Connected	(None, 256), L2, L1 regularization	1
Final	Dropout	(None, 256), rate=0.45	1
Final	Fully Connected	(None, 15), Softmax	1

**Table 3:** Hyperparameters and constants for model training.

Hyperparameter	Value
**Dense Layer Units**	256
**Dense Kernel Regularizer**	L2(0.016)
**Dense Activity Regularizer**	L1(0.006)
**Dense Bias Regularizer**	L1(0.006)
**Dropout Rate**	0.45
**Optimizer**	Adamax
**Optimizer Learning Rate**	0.001
**Loss Function**	Categorical Crossentropy
**Metric**	Accuracy
**Batch Size**	40
**Epochs**	40
**Patience**	1
**Stop Patience**	3
**Threshold**	0.9
**Learning Rate Reduction Factor**	0.5
**Ask Epoch**	5

**Algorithm 1:**
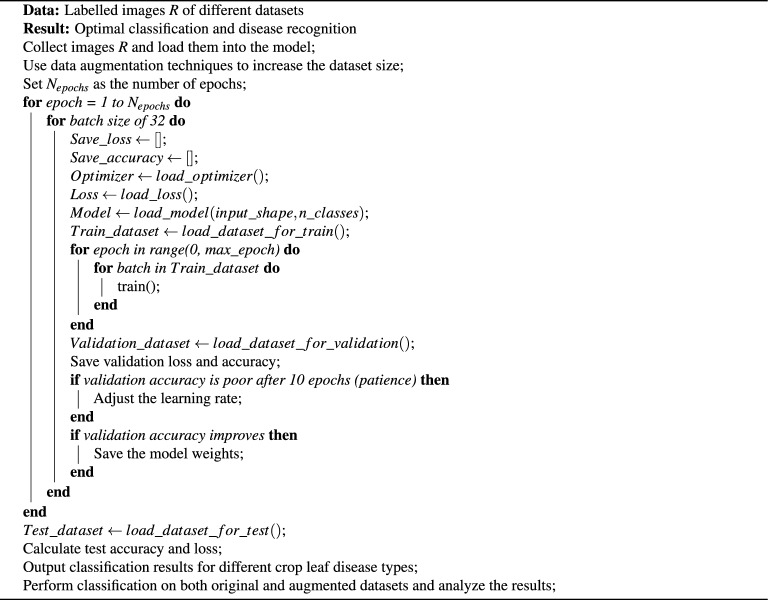
$\hbox {V}^2$PlantNet model algorithm.

## Experimental setup and procedure

The experiments were conducted on Google Colab, utilizing cloud-based resources, including an Intel Xeon CPU @ 2.20GHz, 12.72 GB of RAM, and an NVIDIA T4 GPU, with 358 GB of disk space available for data storage and outputs. The implementation was carried out in Python (version 3.7) with key libraries such as TensorFlow (2.15.0), Keras, NumPy, Pandas, Matplotlib, Seaborn, and OpenCV. Data retrieval was facilitated via Kaggle’s API, providing secure and efficient access to the datasets.

**Fig. 3:**
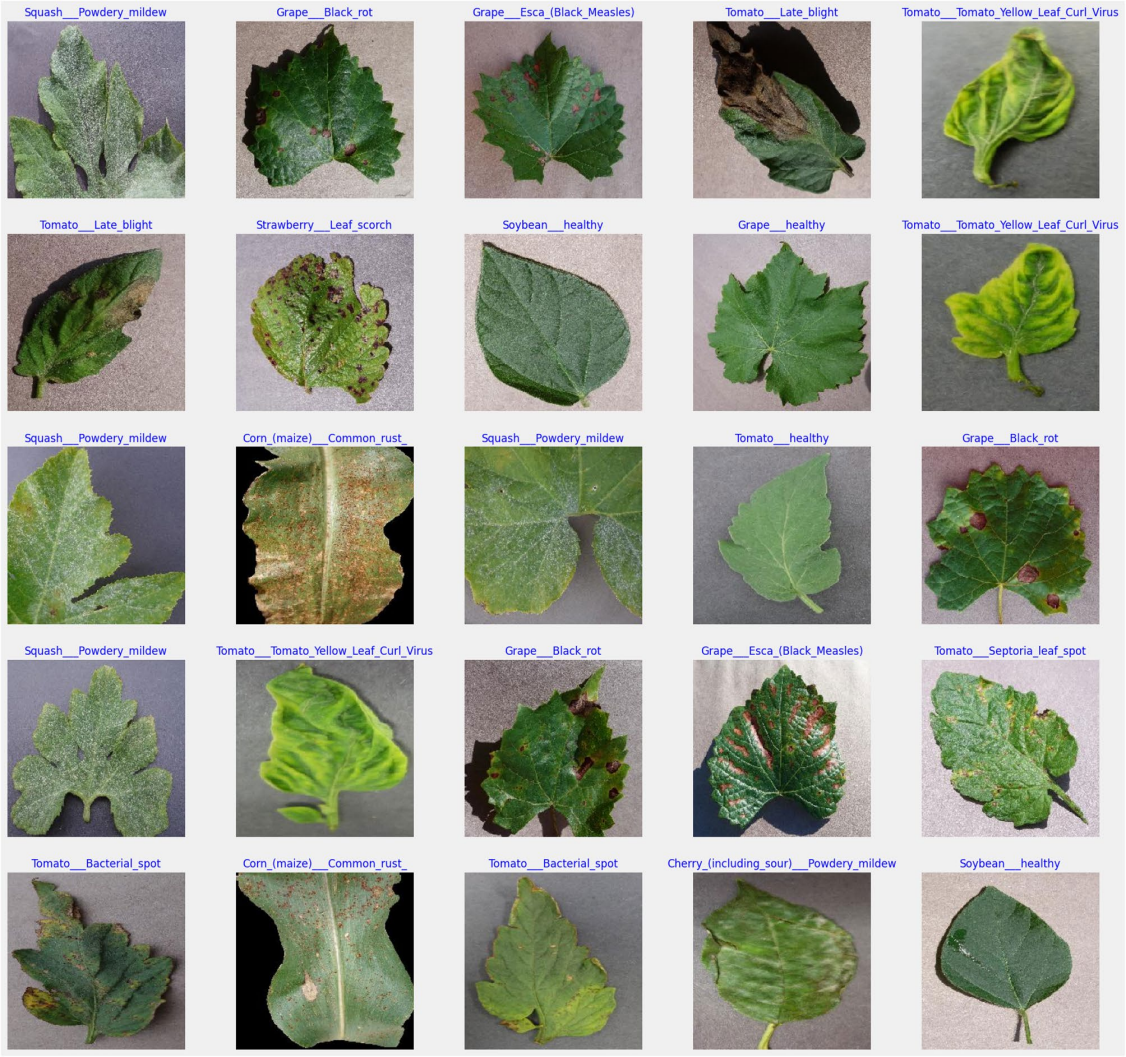
Sample images of Cherry, Corn, Grape, Orange, Peach, Soybean, Squash, Strawberry, and Tomato.

### Data preparation and processing

1. Dataset: The PlantVillage dataset was selected for the experiments, containing 5,431 leaf images across 38 labelled classes, covering both healthy and diseased conditions of 14 different crop types, including Apple, Grape, Corn, Soybean, and Tomato. This dataset was chosen due to its comprehensive coverage of agricultural disease scenarios, providing a diverse training set for robust model development (see link to (dataset)). Sample images from each class are represented in Fig. 3.
2. Data Preprocessing:
  **Normalization:** To standardize the input data, all image pixel values were scaled to the range of 0 to 1 by dividing by 255.0. This normalization step was critical to ensure consistent input scaling, allowing for faster convergence during training and preventing issues related to varying pixel value ranges.
  **Train-validation-test split:** The dataset was split into training (70%), validation (15%), and test (15%) sets. This partitioning ensured that a substantial portion of the data was allocated for model training, while validation and test sets were reserved for evaluating model generalization and performance on unseen data.
  **Class imbalance handling:** Potential class imbalances in the dataset were addressed through dynamic data augmentation techniques, which included horizontal and vertical flips, as well as random adjustments to contrast and brightness. This augmentation helped to artificially increase the variability of the training data, reducing the impact of class imbalance and improving the model’s generalization ability.
3. Data Augmentation: Dynamic data augmentation was applied during training using Keras’ ImageDataGenerator, which ensured real-time data augmentation and efficient tensor conversion. The augmented data was continuously fed into the model, enhancing the diversity of the training set without altering the original images in the validation and test sets. Specifically, we applied transformations such as random rotations, width and height shifts, zooming, shearing, horizontal flips, and rescaling which improved the robustness of the model by exposing it to natural variations in the dataset. To address dataset imbalance, we relied on standard augmentation rather than synthetic image generation. This study did not employ Generative Adversarial Network (GAN)-based or diffusion-based synthesis, as our aim was to preserve the authenticity of real-world plant images and avoid potential artifacts from artificially generated samples. Nonetheless, exploring synthetic data generation remains a promising direction for future work, particularly to improve generalisability in cases of rare or underrepresented species..
4. Data Pipeline: A robust data pipeline was established to manage the loading, preprocessing, and augmentation of images. Data paths were defined, and images were organized into structured data frames to streamline the workflow from data retrieval to model training. This ensured efficient handling of the dataset, from loading images to generating augmented data for training, reducing the computational burden.

The overall procedure for training and evaluating the $\hbox {V}^2$PlantNet model is summarized in Algorithm 1, which outlines the steps involved in data preparation, model training, and performance evaluation. The architectural flow of the $\hbox {V}^2$PlantNet model is illustrated in Fig. 2, providing a visual representation of the data processing stages and model structure.

### Model parameters and performance metrics

The $\hbox {V}^2$PlantNet model was developed and trained using TensorFlow and Keras. Key hyperparameters, such as batch size, learning rate, and the number of epochs, were fine-tuned to optimize model performance, as detailed in Table 3. The Adam optimizer and categorical cross-entropy loss function were employed to ensure effective learning. Adam is widely adopted in computer vision tasks due to its robustness and minimal need for extensive hyperparameter tuning, making it a practical and reliable choice for our model. Dynamic callbacks, including early stopping and learning rate schedulers, were implemented to adjust the learning rate in real-time and prevent overfitting during training. Training history, including loss and accuracy metrics, was logged for detailed analysis.

It is worth noting that although 40 epochs (see Table 3) may appear limited, several factors contributed to the model achieving high accuracy within this range. The input data were carefully normalized and augmented, improving learning efficiency and reducing overfitting. The proposed architecture was designed to extract discriminative features effectively, allowing rapid learning of relevant patterns. In addition, regularization techniques such as dropout, batch normalization, and adaptive learning-rate scheduling enhanced generalization and accelerated convergence. Empirically, validation accuracy plateaued after approximately 15–20 epochs and extending training beyond 40 epochs did not yield meaningful improvements. Thus, training for 40 epochs offered an optimal balance between accuracy and computational efficiency.

The model’s performance was evaluated through various metrics such as accuracy, precision, recall, F1 score, sensitivity, specificity, Matthews Correlation Coefficient (MCC), Average precision (AP), Receiver Operating Characteristic (ROC), and t-Distributed Stochastic Neighbor Embedding (t-SNE). A comparative analysis was performed against state-of-the-art models such as MobileNet, ResNet-50, DenseNet-121, XceptionNet, and EfficientNet, focusing on accuracy, inference time, memory usage, and computational complexity. These metrics were further analyzed using Floating Point Operations (FLOPs), activation counts, and inference time profiling via TensorFlow Profiler.

## Results

### Classification performance

**Fig. 4:**
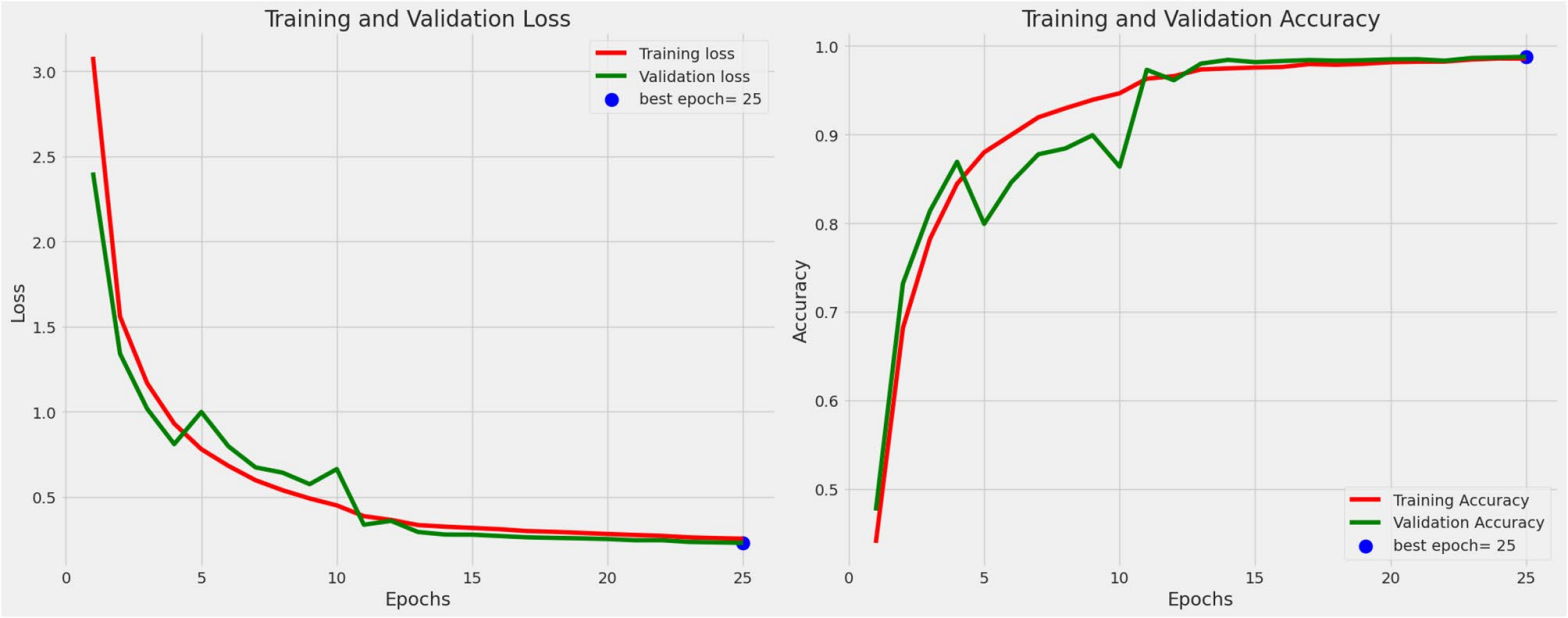
Training and validation loss/accuracy curves.

Figure 4 shows the training and validation loss and accuracy curves, providing insights into the model’s learning dynamics. As illustrated in Fig. 4, the training and validation loss curves exhibit a steep decline during the initial epochs, particularly between epochs 0 and 5, signifying rapid learning. Subsequently, both curves begin to stabilize, with losses gradually decreasing up until around epoch 15. This suggests that the model effectively converges, with minimal overfitting as both training and validation losses closely align. Furthermore, the accuracy curves demonstrate continuous improvement, stabilizing at approximately 98-99% by epoch 20, strengthening the model’s ability to generalize. The optimal epoch was determined to be 25, based on the highest validation accuracy, highlighting the model’s robust performance across diverse datasets.

**Fig. 5:**
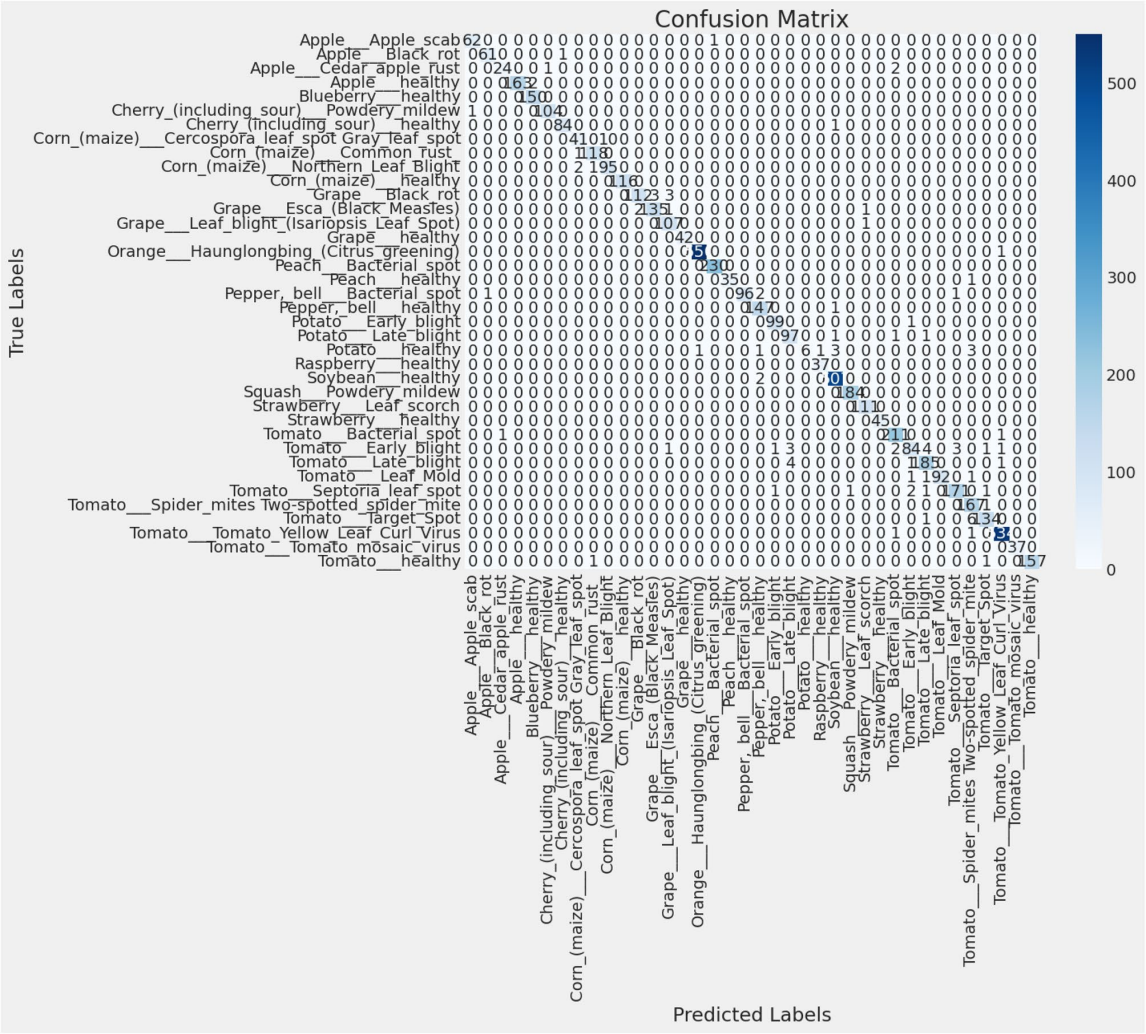
Confusion matrix for the $\hbox {V}^2$PlantNet model on the test dataset.

The performance metrics for the proposed model across various plant disease and health classifications, including Precision, Recall, F1-Score, Sensitivity, Specificity, and MCC, are summarized in Table 4 which shows consistent and high performance across various plant types and diseases. Additionally, Fig. 5 presents the confusion matrices generated for each dataset, where diagonal elements represent correct classifications, and off-diagonal elements highlight misclassifications. These matrices provide valuable insights into the model’s strengths and weaknesses in distinguishing between different classes.

**Table 4:** Performance metrics of the proposed model across various plant disease and health classifications.

Class	Precision	Recall	F1-Score	Sensitivity	Specificity	MCC
Apple (Apple scab)	0.97	0.97	0.97	0.97	0.97	0.97
Apple (Black rot)	1.00	0.98	0.99	0.98	0.98	0.99
Apple (Cedar apple rust)	0.96	0.93	0.94	0.93	0.96	0.94
Apple (healthy)	1.00	0.98	0.99	0.98	1.00	0.99
Blueberry (healthy)	0.99	1.00	0.99	1.00	0.99	0.99
Cherry (including sour) (Powdery mildew)	0.99	1.00	0.99	1.00	0.99	0.99
Cherry (including sour) (healthy)	1.00	0.99	0.99	0.99	0.99	0.99
Corn (maize) (Cercospora leaf spot Gray leaf spot)	0.87	0.80	0.83	0.80	0.93	0.82
Corn (maize) (Common rust)	1.00	1.00	1.00	1.00	1.00	1.00
Corn (maize) (Northern Leaf Blight)	0.88	0.93	0.90	0.93	0.90	0.91
Corn (maize) (healthy)	1.00	1.00	1.00	1.00	1.00	1.00
Grape (Black rot)	0.99	0.99	0.99	0.99	0.99	0.99
Grape (Esca (Black Measles))	1.00	0.99	0.99	0.99	0.98	0.99
Grape (Leaf blight (Isariopsis Leaf Spot))	0.99	1.00	0.99	1.00	0.96	0.97
Grape (healthy)	1.00	1.00	1.00	1.00	1.00	1.00
Orange (Haunglongbing (Citrus greening))	1.00	1.00	1.00	1.00	1.00	1.00
Peach (Bacterial spot)	1.00	0.99	0.99	0.99	1.00	0.99
Peach (healthy)	0.95	0.97	0.96	0.97	1.00	0.96
Pepper, bell (Bacterial spot)	0.97	0.98	0.97	0.98	1.00	0.98
Pepper, bell (healthy)	0.97	0.99	0.98	0.99	0.97	0.98
Potato (Early blight)	1.00	0.99	0.99	0.99	0.98	0.98
Potato (Late blight)	0.97	0.97	0.97	0.97	0.93	0.95
Potato (healthy)	1.00	0.67	0.80	0.67	1.00	0.81
Raspberry (healthy)	1.00	1.00	1.00	1.00	1.00	1.00
Soybean (healthy)	1.00	1.00	1.00	1.00	1.00	1.00
Squash (Powdery mildew)	1.00	0.99	0.99	0.99	0.99	0.99
Strawberry (Leaf scorch)	0.99	1.00	0.99	1.00	0.98	0.99
Strawberry (healthy)	0.98	0.98	0.98	0.98	0.98	0.98
Tomato (Bacterial spot)	0.99	1.00	0.99	1.00	0.97	0.98
Tomato (Early blight)	0.96	0.93	0.94	0.93	0.94	0.93
Tomato (Late blight)	0.97	0.96	0.96	0.96	0.96	0.96
Tomato (Leaf Mold)	0.99	1.00	0.99	1.00	1.00	0.99
Tomato (Septoria leaf spot)	0.98	0.97	0.97	0.97	0.98	0.97
Tomato (Spider mites Two-spotted spider mite)	0.98	1.00	0.99	1.00	0.93	0.96
Tomato (Target Spot)	0.98	0.96	0.97	0.96	0.97	0.96
Tomato (Tomato Yellow Leaf Curl Virus)	1.00	1.00	1.00	1.00	1.00	1.00
Tomato (Tomato mosaic virus)	0.97	1.00	0.98	1.00	1.00	0.98
Tomato (healthy)	0.99	0.99	0.99	0.99	0.99	0.99
**Model Accuracy**				**0.99**		
**Macro avg**	0.98	0.97	0.97	0.97	0.98	0.98
**Weighted avg**	0.99	0.99	0.99	0.99	0.99	0.99

Notably, the model demonstrates high precision and recall scores for several classes, such as ”Apple_Black_rot,” ”Orange-Haunglongbing_(Citrus-greening),” and ”Soybean-healthy,” with both metrics approaching 1.00. This suggests that the model performs exceptionally well in these categories, minimizing false positives and negatives. High specificity and MCC values further support its reliability in these cases, indicating strong overall performance across multiple metrics.

The confusion matrix supports this performance, with most classes showing a strong diagonal presence, reflecting accurate predictions. However, certain classes, such as ”Corn(maize)_Cercospora_leaf_spot” and ”Potato_healthy,” exhibit lower recall scores of 0.80 and 0.67, respectively. This suggests that the model struggles with identifying healthy potato plants, likely due to class imbalance in the training data. The confusion matrix for these classes reveals specific misclassification patterns, highlighting the challenge posed by underrepresented classes.

Class imbalance, particularly in the ”Potato_healthy” category, may contribute to the model’s difficulty, as reflected by a lower MCC. The model also struggles to differentiate between similar diseases within the same plant species, such as those affecting maize or tomatoes, pointing to challenges in feature extraction for subtle intra-class variations. While the model excels in predicting classes with distinct, well-represented features, achieving high F1 scores and strong generalization across most conditions. However, it underperforms in handling underrepresented classes or those with subtle distinctions, where misclassification is more frequent.

**Fig. 6:**
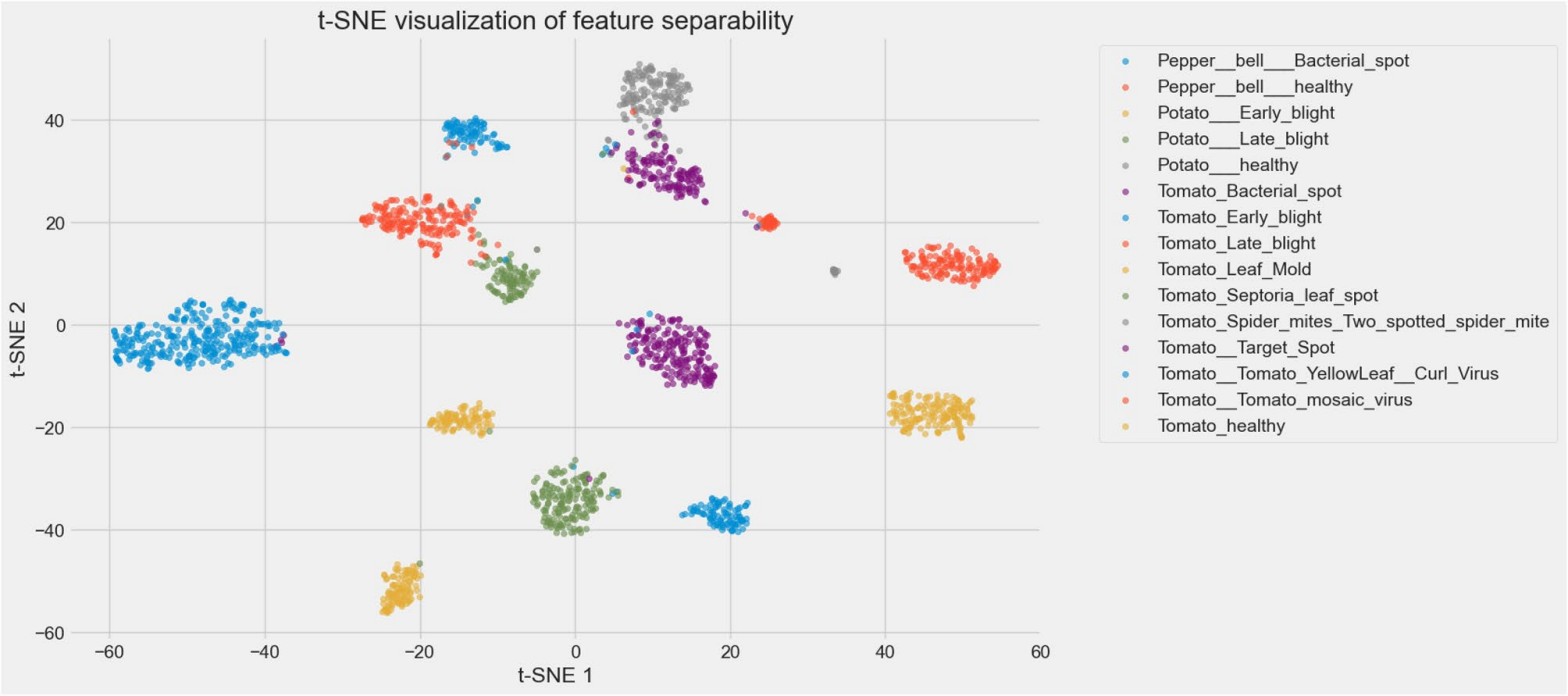
t-SNE visualization of feature embeddings.

**Fig. 7:**
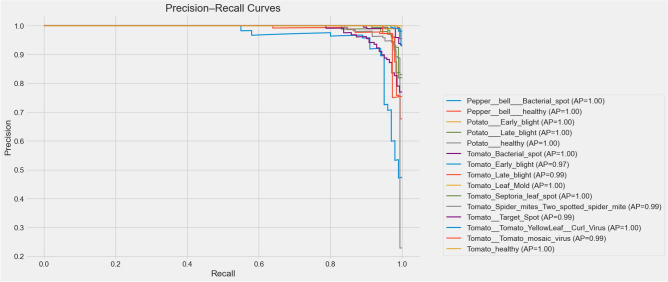
Precision–Recall curves across all classes.

**Fig. 8:**
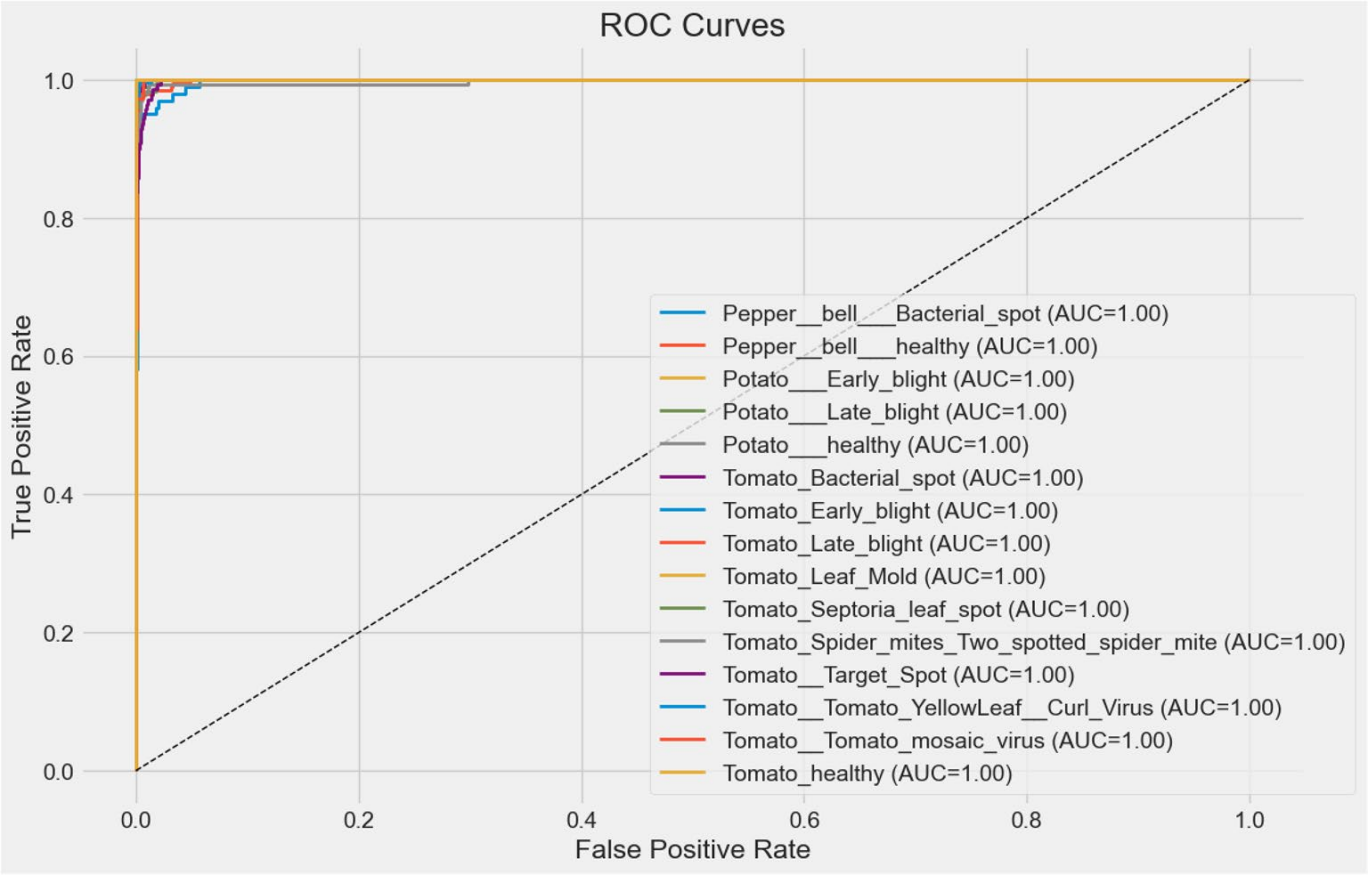
ROC curves across all classes.

Beyond accuracy and loss, the visualization of the t-SNE characteristic in Fig. 6 further emphasizes the discriminative power of the model. By projecting the high-dimensional feature representations into a two-dimensional space, distinct and well-separated clusters corresponding to each of the 16 plant disease classes can be observed. The compactness of intraclass clusters and the clear margins between interclass clusters indicate strong feature separability, confirming that the model has learned meaningful representations capable of distinguishing even visually similar diseases. Furthermore, the precision-recall curves in Fig. 7 reveal consistently high precision and recall values in all classes, with AP ranging from 0.97 to 1.0, highlighting the reliability of the model in minimizing false positives and false negatives. In addition, the ROC curves in Fig. 8 highlight excellent class separability, with almost all classes achieving an area under the curve (AUC) of 1.0. Together, these results, which span training dynamics, feature visualization, and performance metrics, demonstrate that the model is highly robust and generalizable, supporting its potential application in practical real-world agricultural diagnostics.

### Cross-validation performance

**Fig. 9:**
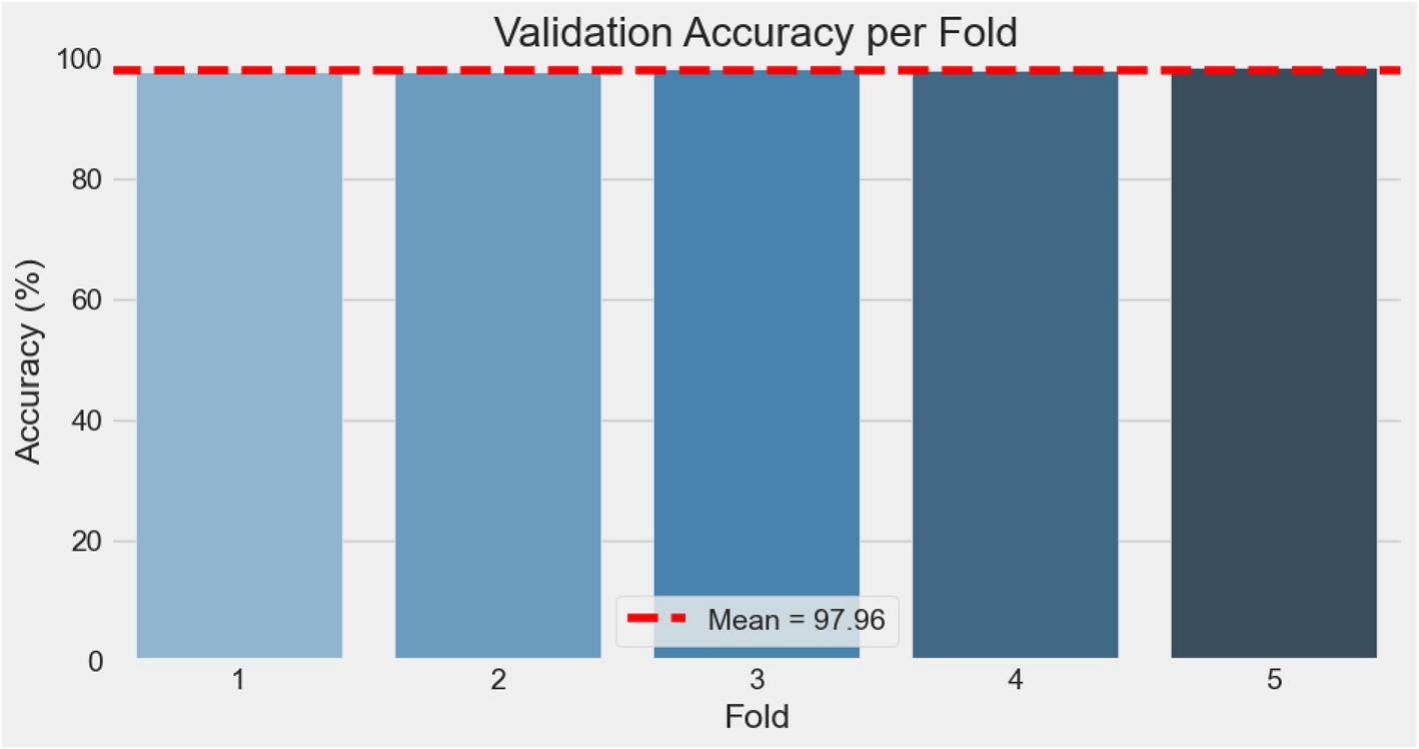
Accuracy per fold.

**Fig. 10:**
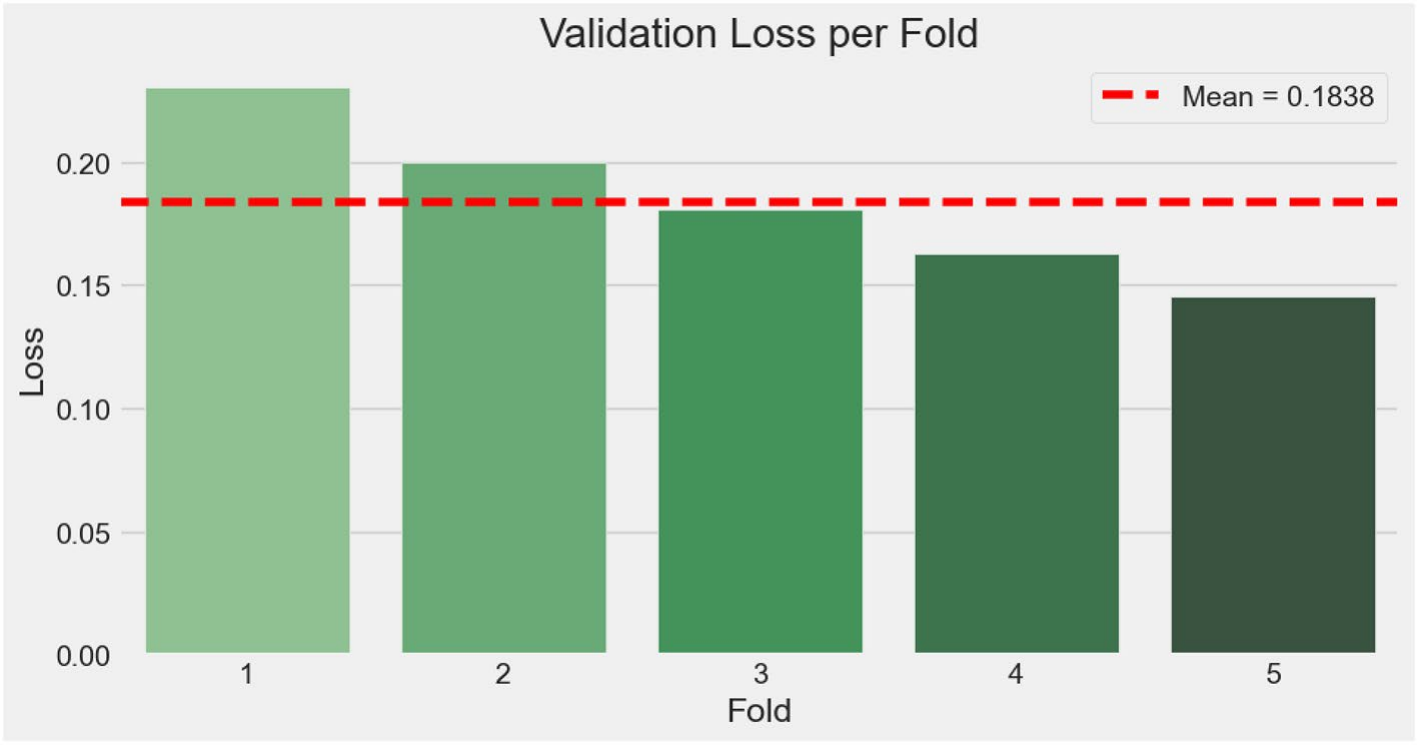
Loss per fold.

**Fig. 11:**
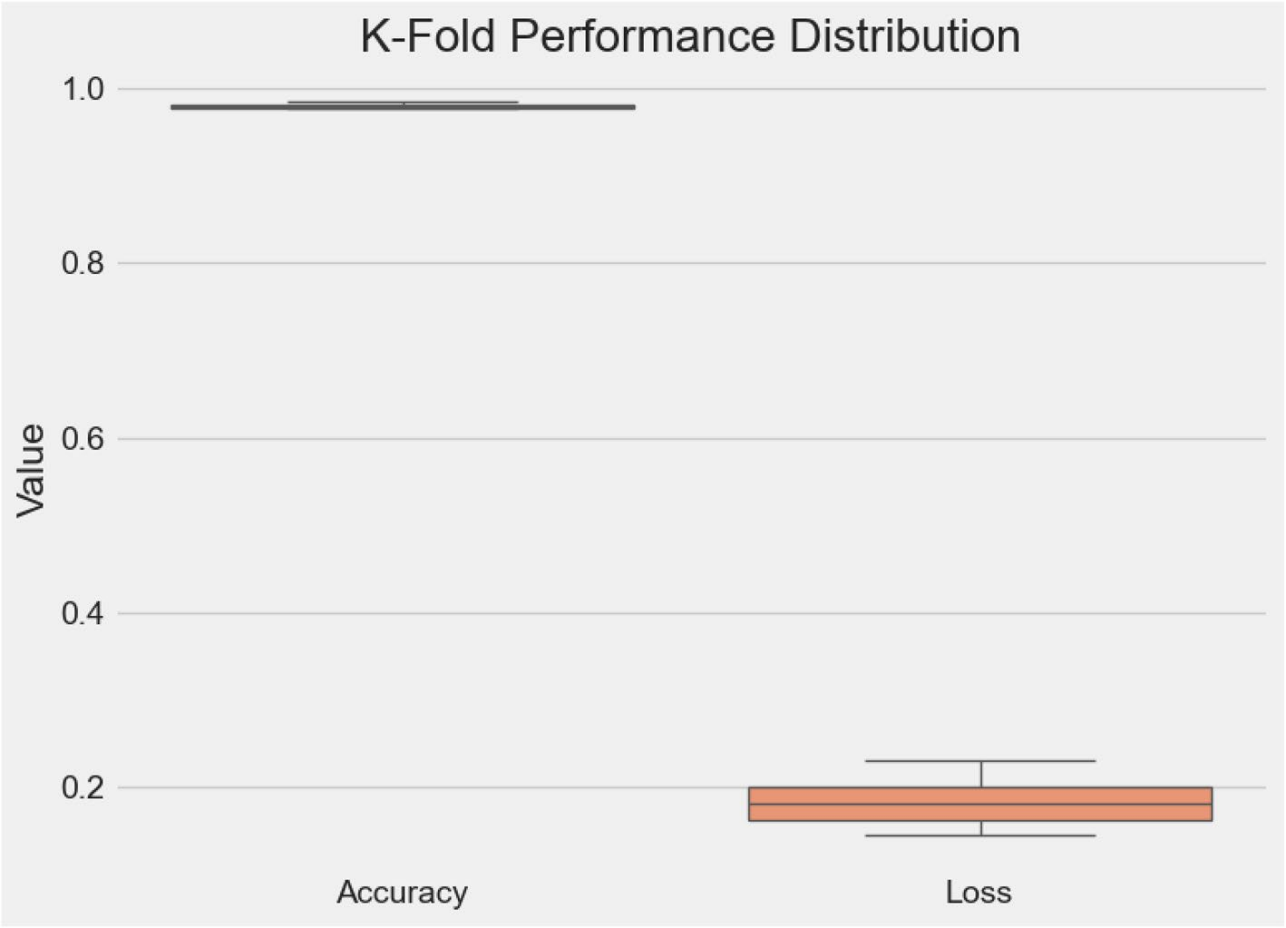
Performance distribution.

To assess model stability and reduce dataset-specific bias, we performed 5-fold cross-validation. Figs. 9, 10, 11 collectively illustrate the fold-wise accuracy, loss, and performance distribution.

**Table 5:** K-fold cross-validation results (5 folds).

Fold	Loss	Accuracy
1	0.2304	0.9767
2	0.1996	0.9777
3	0.1810	0.9806
4	0.1627	0.9791
5	0.1454	0.9840
**Mean**	0.1838	0.9796
**Std. Dev.**	0.0313	0.0025

The results demonstrate that the model maintains consistently high performance across folds, with accuracies ranging from 97.67% to 98.40% and losses between 0.1454 and 0.2304. The average accuracy across folds was 97.96% with a standard deviation of only 0.25%, and the mean loss was 0.1838. The corresponding values are provided in Table 5. These findings highlight the robustness and reliability of $\hbox {V}^2$PlantNet, complementing the single-split evaluation and providing a more comprehensive validation of generalization capability.

### $\hbox {V}^2$PlantNet versus MobileNet

**Table 6:** Performance comparison of $\hbox {V}^2$PlantNet and MobileNet variants on the test dataset.

Model	Total Params	Memory	Inference Time (sec)	TFLOPs	Activations	Precision	Recall	F1-score	Accuracy
$\hbox {V}^2$PlantNet	383,375	1.46 MB	0.676399	773,445	9,659,663	0.99	0.99	0.99	0.99
MobileNet V1	3,244,239	12.38 MB	0.758443	6,488,588	16,843,215	0.99	0.99	0.99	0.99
MobileNet V2	2,277,199	8.69 MB	1.361673	4,554,633	21,814,975	1.0	1.0	1.0	1.0
MobileNet V3 Large	3,010,767	11.49 MB	1.343	6,011,595	19,962,943	0.99	0.99	0.99	0.99
MobileNet V3 Small	947,775	3.62 MB	1.812190	1,889,937	7,755,103	1.00	1.00	1.00	1.00

Table 6 provides an in-depth comparison of the $\hbox {V}^2$PlantNet model against various MobileNet versions, examining multiple performance metrics such as parameter count, memory consumption, inference time, FLOPs, activations, and classification performance. According to this table, the $\hbox {V}^2$PlantNet model, which comprises only 383,375 parameters and requires 1.46 MB of memory, achieves an inference time of 0.676 seconds with 773,445 FLOPs. Despite its minimalistic structure, $\hbox {V}^2$PlantNet achieves remarkable classification performance, with weighted average precision, recall, F1-score, and accuracy all reaching 0.99, indicating an efficient balance between computational demands and accuracy.

In contrast, Table 6 shows that MobileNet V1, with 3,244,239 parameters and a memory footprint of 12.38 MB, has a longer inference time of 0.758 seconds and requires 6,488,588 FLOPs. It achieves weighted averages of 0.99 for precision, recall, F1-score, and accuracy, matching $\hbox {V}^2$PlantNet’s classification performance. MobileNet V2, containing 2,277,199 parameters and requiring 8.69 MB of memory, records an even longer inference time of 1.362 seconds and 4,554,633 FLOPs, although it achieves perfect scores (1.0) across all classification metrics. MobileNet V3 Large, which has 3,010,767 parameters and a memory usage of 11.49 MB, records an inference time of 1.343 seconds and 6,011,595 FLOPs, with weighted averages of 0.99 for precision, recall, F1-score, and accuracy, comparable to both the $\hbox {V}^2$PlantNet model and MobileNet V1. However, MobileNet V3 Small records the longest inference time at 1.812 seconds, despite its parameter-efficient architecture with 947,775 parameters and 3.62 MB of memory usage

The longer inference time of MobileNet V3 Small despite the comparatively low parameter count, FLOPs, and number of activations can be attributed to several factors. Its architecture may include complex operations that require more computation for each parameter, leading to increased processing time. Additionally, certain features or layers in its design may add extra steps, further slowing down its speed. While the model is optimized to use fewer parameters, which makes it smaller and more efficient, this emphasis on efficiency can sometimes come at the cost of speed. Moreover, if MobileNet V3 Small is less optimized for batch processing, it could experience further delays. Despite achieving perfect classification metrics (1.0 for precision, recall, F1-score, and accuracy), its slower processing time underscores the trade-offs between performance and efficiency. In summary, the proposed $\hbox {V}^2$PlantNet model achieves an effective balance between parameter efficiency and computational performance. While MobileNet V2 and MobileNet V3 Small exhibit marginally superior classification metrics, the $\hbox {V}^2$PlantNet model remains a highly competitive option, providing a more parameter-efficient alternative compared to the other models assessed.

### Ablation study

**Table 7:** Ablation study results for $\hbox {V}^2$PlantNet model.

Ablation Study	Test Accuracy (%)	Train Accuracy (%)	Validation Accuracy (%)	Validation Loss	Epochs
Batch Normalization Removed	10.15	10.14	10.14	3.380	5
Dropout Removed	98.38	99.61	98.36	0.226	Early Convergence
Regularization Removed	98.53	99.73	98.38	0.050	Early Convergence

Table 7 presents the results from three ablation studies conducted on the $\hbox {V}^2$PlantNet model, aimed at evaluating the impact of Batch Normalization (BN) , Dropout, and Regularization on model performance. By systematically removing these components, we assessed their influence on accuracy, generalization, and computational efficiency.

The first ablation study examined the removal of BN layers, which stabilize the learning process by normalizing inputs to each layer. Removing BNlayers drastically reduced test accuracy to 10.15%, with training accuracy similarly low at 10.14%. Both training and validation accuracies stagnated, and early stopping was triggered at epoch 5 due to the model’s inability to improve on validation data. The findings indicate that BN layers are critical for effective learning, as their absence resulted in poor convergence and generalization, evidenced by a high validation loss of 3.38.

The second ablation study focused on the removal of Dropout layers, typically used to prevent overfitting. Surprisingly, the model’s test accuracy improved to 98.38% without Dropout, suggesting that Dropout may have introduced unnecessary regularization in this case. The model achieved early training convergence, with a train accuracy of 99.61%, validation accuracy of 98.36%, and validation loss of 0.226. This study highlights that Dropout’s effectiveness is context-dependent, and in this scenario, its removal enhanced the model’s learning capacity.

The third study evaluated the effect of removing Regularization (e.g., L2 regularization), which typically controls overfitting by penalizing large model parameters. Without regularization, the model achieved a test accuracy of 98.53%, slightly higher than when Dropout was removed. Train accuracy reached 99.73%, and validation accuracy was 98.38%, with a lower validation loss of 0.050. These results suggest that the model was robust enough to generalize well without regularization, likely due to the stability of its architecture.

The ablation studies provided key insights into the $\hbox {V}^2$PlantNet model’s architecture, highlighting BN as essential for stabilizing training and ensuring convergence, while Dropout and Regularization, though useful, were less critical in this context. These findings suggest that future model designs can benefit from selectively omitting certain components, like Dropout or Regularization, depending on the task and dataset. Overall, these insights underscore the importance of architectural modifications in shaping the model’s learning dynamics, offering a foundation for optimizing both computational efficiency and performance.

### Comparative performance analysis

To put these findings into context, a comparative analysis was performed against several state-of-the-art models, including XceptionNet, DenseNet-121, ResNet-50, VGG-16, AlexNet, EfficientNet B2, and SoyNet. The performance metrics for these models are based on experiments conducted by Sharma et al. (2023)^12^ and others, who used datasets different from those employed for the $\hbox {V}^2$PlantNet Model. As such, while the comparisons provide useful insights, caution should be exercised when interpreting the results due to probable variances in dataset properties. The detailed comparisons and performance metrics are summarized in the tables below, illustrating both the strengths and limitations of the proposed model relative to other models.

**Table 8:** Model comparisons on parameter count.

Model Name	Trainable Parameters	Non Trainable Parameters	Total Parameters
Xception Network^12^	20,815,148	54,528	20,869,676
Densenet-121^12^	6,966,020	81,472	7,047,492
Alexnet^12^	46,764,612	1,216	46,765,828
VGG-16^12^	107,038,538	0	107,038,538
Resnet-50^12^	23,542,788	53,120	23,595,908
EfficientNet B2^12^	7,706,630	67,575	7,774,205
SoyNet Model^12^	1,338,148	0	1,338,148
**V**$^2$**PlantNet**	**382,246**	**7,040**	**389,286**

Table 8 provides a comparison of trainable and non-trainable parameters across various deep learning models, showcasing the balance between model complexity and computational efficiency. Among the models, XceptionNet stands out as the most computationally intensive, with 20,869,676 parameters. DenseNet-121, with 7,047,492 parameters, is more efficient but still maintains a sizable parameter count, though significantly lower than XceptionNet and AlexNet. AlexNet and VGG-16, requiring 46,765,828 and 107,038,538 parameters respectively, are large models that demand substantial computational resources and extended training times. In contrast, ResNet-50 strikes a more balanced approach with 23,595,908 parameters, optimizing both parameter efficiency and architectural complexity. EfficientNet B2, with 7,774,205 parameters, prioritizes computational efficiency while maintaining strong performance, reflecting a well-balanced design. SoyNet, with only 1,338,148 parameters, is an ultra-lightweight model, though its reduced complexity may limit its performance. The $\hbox {V}^2$PlantNet model stands out with an extremely low parameter count of 389,286, including 382,246 trainable and 7,040 non-trainable parameters. This count is comparable to SoyNet, yet $\hbox {V}^2$PlantNet achieves this efficiency without sacrificing classification performance, making it highly suitable for resource-constrained environments. Compared to larger models such as VGG-16 and AlexNet, $\hbox {V}^2$PlantNet provides a compelling alternative, combining computational efficiency with competitive accuracy, and thus offering an advantage in scenarios where both performance and resource optimization are key.

Table 9 compares the performance of the $\hbox {V}^2$PlantNet Model with other models on the grape dataset. The results demonstrate that the $\hbox {V}^2$PlantNet Model outperforms the others, achieving near-perfect test accuracy (up to 99.94%) and flawless precision, recall, sensitivity, and specificity for the Healthy class. For other classes, such as Esca, Black Rot, and Leaf Blight, the model consistently delivered high performance, with precision, recall, and F1-scores exceeding 0.95. The XceptionNet also exhibited strong performance, with test accuracies ranging from 91.00% to 100.00%, particularly excelling in the Healthy class, though with slightly lower metrics in other classes. DenseNet-121 achieved near-perfect results across all classes, with a test accuracy of up to 99.22% for Leaf Blight. AlexNet performed competitively, especially in the Healthy and Black Rot classes, though its large parameter size could hinder deployment in some cases. VGG-16 showed variable performance, performing best on Leaf Blight but underperforming in other classes, while ResNet-50 had lower performance, particularly in Black Rot. EfficientNet B2 exhibited highly variable performance, with test accuracies ranging from 20.0% to 76.17%, and SoyNet, while generally performing well, had slightly reduced performance in the Esca and Black Rot classes. These findings highlight the robust classification capabilities of the $\hbox {V}^2$PlantNet Model across various grape disease categories, establishing it as an effective tool for plant leaf disease classification on the grape dataset.

**Table 9:** Model Comparisons on the grapes dataset.

Model Name	Class	Train Acc.	Test Acc.	Precision	Recall	Sensitivity	Specificity	F1	MCC
**Xception Network**	Healthy	99.04	95.34	1.00	0.99	0.99	1.00	0.99	0.99
	Esca	97.44		0.91	0.96	0.96	0.96	0.92	0.92
	Black rot	93.33		0.91	0.95	0.95	0.96	0.92	0.92
	Leaf Blight	95.94		0.89	0.93	0.93	0.92	0.89	0.89
**Densenet-121**	Healthy	98.88	96.91	0.98	0.97	0.97	0.98	0.97	0.97
	Esca	98.50		0.92	0.97	0.97	0.97	0.93	0.93
	Black rot	98.50		0.92	0.96	0.96	0.97	0.93	0.93
	Leaf Blight	99.22		0.98	0.98	0.98	0.99	0.98	0.98
**Alexnet**	Healthy	99.58	97.84	0.98	0.98	0.98	0.99	0.98	0.97
	Esca	98.79		0.98	0.98	0.98	0.99	0.98	0.98
	Black rot	98.89		0.97	0.98	0.98	0.99	0.97	0.97
	Leaf Blight	98.66		0.97	0.98	0.98	0.99	0.98	0.97
**VGG-16**	Healthy	97.06	89.75	0.97	0.89	0.89	0.99	0.93	0.91
	Esca	93.19		0.83	0.91	0.91	0.93	0.87	0.83
	Black rot	90.58		0.84	0.78	0.78	0.94	0.81	0.76
	Leaf Blight	98.83		0.95	0.99	0.99	0.98	0.97	0.96
**Resnet-50**	Healthy	82.43	45.65	0.96	0.26	0.26	0.99	0.40	0.45
	Esca	74.93		0.51	0.89	0.89	0.69	0.65	0.55
	Black rot	57.22		0.32	0.57	0.57	0.57	0.41	0.28
	Leaf Blight	76.73		0.91	0.02	0.02	0.99	0.04	0.13
**EfficientNet B2**	Healthy	76.56	26.59	0	0	0	1		
	Esca	26.59		0.26	1	1	0	0.42	
	Black rot	73.85		0	0	0	1		
	Leaf Blight	76.17		0	0	0	1		
**SoyNet Model**	Healthy	99.22	93.30	0.98	0.98	0.98	0.99	0.98	0.97
	Esca	94.95		0.94	0.85	0.85	0.98	0.90	0.87
	Black rot	93.51		0.84	0.92	0.92	0.93	0.88	0.84
	Leaf Blight	98.89		0.98	0.97	0.97	0.99	0.97	0.96
**V**$^2$**PlantNet**	Healthy	99.88	99.50	1.00	1.00	1.00	1.00	1.00	1.00
	Esca	99.50		1.00	0.99	0.99	0.98	0.99	0.99
	Black rot	99.66		0.99	0.99	0.99	0.99	0.99	0.99
	Leaf Blight	99.94		0.99	1.00	1.00	0.96	0.99	0.97

**Table 10:** Model comparisons on the tomato dataset.

Model Name	Class	Train Acc.	Test Acc.	Precision	Recall	Sensitivity	Specificity	F1	MCC
**Xception Network**	Bacterial Spot	99.38	94.13	0.95	0.97	0.97	0.99	0.96	0.96
	Healthy	99.49		0.98	0.97	0.97	0.99	0.97	0.97
	Septoria Spot	99.21		0.98	0.93	0.93	0.99	0.95	0.95
	Mosaic Virus	99.80		0.98	0.99	0.99	0.99	0.98	0.98
	Target Spot	97.66		0.87	0.89	0.89	0.98	0.88	0.87
	Early Blight	97.57		0.84	0.93	0.93	0.98	0.88	0.87
	Spider Mites	98.58		0.92	0.92	0.92	0.99	0.92	0.91
	Late Blight	97.92		0.92	0.85	0.85	0.99	0.88	0.88
	Yellow Curl	99.28		0.98	0.94	0.94	0.99	0.96	0.96
	Leaf Mold	99.32		0.95	0.97	0.97	0.99	0.96	0.96
**Densenet-121**	Bacterial Spot	99.21	94.90	0.94	0.97	0.97	0.99	0.95	0.95
	Healthy	99.67		0.98	0.98	0.98	0.99	0.98	0.98
	Septoria Spot	99.12		0.96	0.94	0.94	0.99	0.95	0.94
	Mosaic Virus	99.62		0.97	0.98	0.98	0.99	0.97	0.97
	Target Spot	98.18		0.90	0.91	0.91	0.98	0.90	0.90
	Early Blight	98.18		0.90	0.92	0.92	0.98	0.91	0.90
	Spider Mites	98.58		0.92	0.93	0.93	0.99	0.92	0.91
	Late Blight	98.34		0.93	0.89	0.89	0.99	0.91	0.90
	Yellow Curl	99.49		0.98	0.96	0.96	0.99	0.97	0.97
	Leaf Mold	99.34		0.96	0.97	0.97	0.99	0.96	0.96
**Alexnet**	Bacterial Spot	99.10	90.84	0.94	0.95	0.95	0.99	0.95	0.94
	Healthy	99.30		0.95	0.97	0.97	0.99	0.96	0.96
	Septoria Spot	97.51		0.89	0.83	0.83	0.98	0.86	0.85
	Mosaic Virus	99.58		0.96	0.99	0.99	0.99	0.97	0.97
	Target Spot	97.01		0.84	0.85	0.85	0.98	0.85	0.83
	Early Blight	96.22		0.80	0.84	0.84	0.97	0.82	0.80
	Spider Mites	97.73		0.87	0.88	0.88	0.98	0.88	0.86
	Late Blight	97.77		0.91	0.86	0.86	0.99	0.88	0.87
	Yellow Curl	99.19		0.95	0.96	0.96	0.99	0.96	0.95
	Leaf Mold	98.23		0.92	0.90	0.90	0.99	0.91	0.90

Model Name	Class	Train Acc.	Test Acc.	Precision	Recall	Sensitivity	Specificity	F1	MCC
**Resnet-50**	Bacterial Spot	90.57	50.13	0.34	0.01	0.01	0.99	0.03	0.07
	Healthy	84.75		0.13	0.08	0.08	0.93	0.10	0.09
	Septoria Spot	90.14		0.10	0.01	0.01	0.99	0.01	0.02
	Mosaic Virus	80.52		0.17	0.25	0.25	0.86	0.20	0.18
	Target Spot	75.31		0.17	0.39	0.39	0.79	0.24	0.22
	Early Blight	84.47		0.12	0.07	0.07	0.93	0.09	0.08
	Spider Mites	84.01		0.15	0.15	0.15	0.91	0.15	0.13
	Late Blight	85.49		0.12	0.07	0.07	0.94	0.09	0.08
	Yellow Curl	71.73		0.22	0.64	0.64	0.72	0.32	0.31
	Leaf Mold	88.17		0.10	0.01	0.01	0.98	0.03	0.04
**SoyNet Model**	Bacterial Spot	94.52	69.14	0.66	0.81	0.81	0.95	0.73	0.71
	Healthy	97.07		0.88	0.82	0.82	0.98	0.85	0.84
	Septoria Spot	91.75		0.56	0.56	0.56	0.95	0.56	0.53
	Mosaic Virus	96.40		0.78	0.86	0.86	0.97	0.82	0.80
	Target Spot	93.17		0.64	0.71	0.71	0.95	0.67	0.65
	Early Blight	90.75		0.56	0.52	0.52	0.95	0.54	0.51
	Spider Mites	91.97		0.57	0.60	0.60	0.95	0.58	0.56
	Late Blight	92.86		0.69	0.52	0.52	0.97	0.59	0.58
	Yellow Curl	96.00		0.80	0.82	0.82	0.97	0.81	0.79
	Leaf Mold	93.74		0.71	0.64	0.64	0.97	0.67	0.65
**V**$^2$**PlantNet**	Bacterial Spot	99.00	98.00	0.97	0.99	0.98	0.99	0.98	0.98
	Healthy	99.00		1.00	0.99	0.99	0.99	0.99	0.99
	Septoria Spot	99.00		0.98	0.97	0.97	0.99	0.97	0.97
	Mosaic Virus	99.00		1.00	1.00	1.00	0.99	1.00	1.00
	Target Spot	99.00		0.97	0.95	0.96	0.98	0.96	0.96
	Early Blight	99.00		0.94	0.84	0.84	0.98	0.89	0.89
	Spider Mites	99.00		0.93	0.99	0.96	0.98	0.96	0.96
	Late Blight	99.00		0.96	0.97	0.97	0.98	0.96	0.96
	Yellow Curl	99.00		0.99	1.00	0.99	0.99	0.99	0.99
	Leaf Mold	99.00		1.00	0.97	0.98	0.98	0.98	0.98

In addition to the performance metrics presented for grape disease classification, Table 10 offers a comparative analysis of multiple models on the Tomato dataset. The proposed model outperformed others across all categories, achieving nearly perfect or flawless test accuracies and classification metrics. Specifically, it delivered test accuracies of 99.0% for all classes, along with impeccable precision, recall, F1-scores, and specificity in most categories. This consistently high performance highlights the model’s robustness and adaptability to a wide range of tomato disease types, positioning it as an outstanding solution for this dataset.

In comparison, the Xception Network and DenseNet-121 also performed well, achieving test accuracies of up to 99.80% and 99.67%, respectively, but their performance showed greater variability across different disease classes. For example, Xception Network delivered excellent results with a test accuracy of 99.80% for Mosaic Virus and 99.49% for Healthy, but performed less consistently in classes like Target Spot and Early Blight. Similarly, DenseNet-121 achieved high accuracy, reaching 99.67% for Healthy, but encountered difficulties with Target Spot and Early Blight, revealing slight inconsistencies in handling specific disease categories. AlexNet, ResNet-50, and SoyNet exhibited even broader variations in performance, with significant discrepancies in accuracy and precision across different classes, further emphasizing the exceptional ability of the proposed model to maintain consistently high performance across various tomato disease categories.

**Table 11:** Comparison of classification models based on parameters and accuracy scores.

Researchs	Dataset	Model	Accuracy (%)	Params
Arsenovic et al. (2019)^38^	Real-time images	PlantDiseaseNet	93.67	21.4 Million
Mohameth et al. (2020)^39^	Plant Village	ResNet-50 + SVM	95.38	25 Million
Chen et al. (2020)^40^	Plant Village	INC-VGGN	91.83	41 Million
Argueso et al. (2020)^41^	Plant Village	Inception V3	91.40	24 Million
Nanehkaran et al. (2020)^42^	Plant Village (Rice, Maize, Potato)	K means with CNN	75.59	8.9 Million
Khamparia et al. (2020)^43^	Plant Village (Maize, Potato)	Convolutional encoder network	86.78	3.3 Million
Zhao et al. (2022)^44^	Plant Village (Corn, Potato, Tomato)	RIC-Net (Residual inception network)	95.20	6.71 Million
Turkoglu et al. (2022)^45^	TurkeyPlantDataset	PlantDiseaseNet	96.00	9.1 Million
Bao et al. (2021)^22^	Real-time (Wheat ear)	SimpleNet	94.1	2.3 Million
Sharma et al. (2023)^12^	Plant Village	DLMNC-Net	93.56 to 99.50	6.4 Million
**Proposed research**	**Plant Village and Kaggle**	**V**$^2$**PlantNet**	**99.0**	**389,286 only**

A comparative analysis on the classification performance among different recent methods are provided in Table 11. As illustrated in this table, $\hbox {V}^2$PlantNet consistently delivers competitive performance compared to several state-of-the-art models, excelling in both accuracy and computational efficiency.

The study by Arsenovic et al.^38^ developed PlantDiseaseNet, a model that achieved 93.67% accuracy while utilizing 21.4 million parameters. Although PlantDiseaseNet demonstrated strong accuracy, its substantial parameter count of 21.4 million limits its practicality for real-time applications in resource-constrained environments. The high computational demands make it less suitable for systems with restricted processing power or memory capacity. In contrast, $\hbox {V}^2$PlantNet achieved a remarkable accuracy of 99% using only 389,286 parameters, underscoring its scalability and efficiency. Similarly, Mohameth et al.^39^ combined ResNet-50 with an SVM classifier, obtaining an accuracy of 95.38% but with 25 million parameters. While ResNet-50 remains popular for its robust performance, the computational burden it imposes is a significant limitation. $\hbox {V}^2$PlantNet, with fewer parameters and better accuracy, emerges as a more optimal solution.

In another study, Chen et al.^40^ introduced the INC-VGGN model, which employed an ensemble approach and achieved 91.83% accuracy. However, this model required a substantial 41 million parameters. Despite its innovative design, the large parameter count limits its efficiency, especially when compared to the compact design of $\hbox {V}^2$PlantNet, which delivers superior performance with a fraction of the parameters. Similarly, a separate study by Argüeso et al.^41^ utilized transfer learning with the Inception V3 model, achieving an accuracy of 91.40%, accuracy while requiring 24 million parameters. Although the Inception V3 is known for its versatility, its heavy-parameter architecture limits its application in low-resource environments. The proposed $\hbox {V}^2$PlantNet model, through careful architectural optimization, not only achieves better accuracy but also dramatically reduces computational demands.

Additionally, the study by Nanehkaran et al. used a mixture of K-means clustering and CNN to achieve 75.59% accuracy with 8.9 million parameters^42^. This relatively low accuracy, combined with a higher parameter count, demonstrates the limitations of this approach. In contrast, the study by Khamparia et al.^43^ developed a Convolutional Encoder Network, achieving 86.78% accuracy with 3.3 million parameters. While more lightweight, this model’s accuracy still falls short of $\hbox {V}^2$PlantNet, which delivers higher accuracy with a comparable parameter count.

Further comparative analysis includes the work of Zhao et al.^44^, who developed RIC-Net, achieving 95.20% accuracy with 6.71 million parameters. While RIC-Net utilizes inception modules and residual blocks effectively, it still requires more parameters than $\hbox {V}^2$PlantNet, which achieves higher accuracy with considerably fewer computational resources. Similarly, the study by Turkoglu et al.^45^ adapted PlantDiseaseNet for the TurkeyPlantDataset, achieving a 96.00% accuracy with 9.1 million parameters, once again highlighting $\hbox {V}^2$PlantNet’s superior parameter efficiency.

Further study by Bao et al.^22^ introduced the SimpleNet model. The SimpleNet as an efficient CNN model achieved 94.1% accuracy with 2.13 million parameters. $\hbox {V}^2$PlantNet surpasses it by achieving higher accuracy with an even smaller parameter count. Finally, Sharma et al.^12^ introduced DLMNC-Net, with accuracies ranging from 93.56% to 99.50% and 6.4 million parameters. Although DLMNC-Net achieves near-perfect accuracy, $\hbox {V}^2$PlantNet matches this performance with significantly fewer parameters, positioning it as a more computationally efficient solution.

Overall, the proposed $\hbox {V}^2$PlantNet model demonstrates exceptional performance in both accuracy and computational efficiency. The model’s ability to maintain high classification accuracy across diverse datasets with minimal computational resources makes it a promising solution for real-time, resource-constrained applications. The comparison of $\hbox {V}^2$PlantNet with other models underscores the usefulness of lightweight architectures to balance performance and resource efficiency, paving the way for more scalable deep learning models in agricultural diagnostics.

### Limitations and future work

A key limitation of this work lies in the *black-box nature of deep learning models*, which constrains interpretability and may hinder user trust. Although quantitative metrics and visualization techniques (t-SNE, ROC, precision–recall) confirm robustness, they do not explain the underlying decision-making process, which is an essential requirement for practical agricultural adoption.

Future work should therefore integrate *Explainable AI (XAI)* techniques such as Grad-CAM, LIME, and SHAP to provide visual and quantitative insights into model behavior, ensuring that predictions are grounded in biologically relevant features. Recent studies confirm their value in crop disease detection, including tomato^46^ and corn^47^. Beyond single-model strategies, combining XAI with lightweight and ensemble approaches offers further promise. Rashid et al.^48^ achieved 99% accuracy in cucumber disease detection with an XAI-enabled ensemble suitable for edge deployment, while Ajay et al.^49^ proposed LeafVisionNet, a lightweight attention-based model that surpassed 99% accuracy with improved interpretability. Similarly, Senthil et al.^50^ demonstrated the potential of integrating AI with IoT frameworks for real-time optimization in hydroponics, achieving 99.1% accuracy and 97.3% recall.

Building on these advances, future extensions of $\hbox {V}^2$PlantNet should integrate XAI for interpretability, explore lightweight ensemble strategies for adaptability, and leverage IoT connectivity for real-time monitoring. Such enhancements will help transform $\hbox {V}^2$PlantNet into a reliable, transparent, and field-ready diagnostic tool for sustainable agriculture.

## Conclusion

This paper presents $\hbox {V}^2$PlantNet, a novel, lightweight CNN architecture designed for multi-class plant disease classification. The model builds on the MobileNet V1 architecture by incorporating BN and ReLU activation after each convolutional layer and adopting a multi-stage approach to enhance performance. $\hbox {V}^2$PlantNet was evaluated using a comprehensive dataset comprising 38 disease classes in 14 types of crops. The model achieved a high classification accuracy of 99%, along with impressive performance metrics such as precision, recall, F1 score, sensitivity, specificity, and MCC, while using only 389,286 parameters.

Beyond accuracy, visualization of the t-SNE characteristics revealed clear separability between classes, while precision recall and ROC curves further confirmed the reliability of the model between classes. To validate generalization and reduce dataset-specific bias, 5-fold cross-validation demonstrated stable performance, with an average accuracy of 97.96% and a standard deviation of 0.25%, highlighting robustness and consistency. Together, these results confirm the potential of $\hbox {V}^2$PlantNet as a scalable and reliable solution for the detection of plant disease in real time.

## Data Availability

The datasets generated and analysed during the current study are available in the Kaggle repository (see link to dataset: https://www.kaggle.com/datasets/abdallahalidev/plantvillage-dataset).

## References

[1] Wegren, S. K. & Elvestad, C. Russia’s food self-sufficiency and food security: An assessment. *Post-Communist Econ.* **30**, 565–587 (2018).

[2] Employment indicators 2000–2023 – july 2025 update. FAOSTAT Analytical Briefs, No. 110 (2025). Rome. 10.4060/cd5821en.

[3] Pawlak, K. & Kołodziejczak, M. The role of agriculture in ensuring food security in developing countries: Considerations in the context of the problem of sustainable food production. *Sustainability* **12**, 5488 (2020).

[4] Prosekov, A. Y. & Ivanova, S. A. Food security: The challenge of the present. *Geoforum* **91**, 73–77 (2018).

[5] Savary, S. et al. The global burden of pathogens and pests on major food crops. *Nat. ecology & evolution* **3**, 430–439 (2019). 30718852 10.1038/s41559-018-0793-y

[6] Kashyap, B. & Kumar, R. Sensing methodologies in agriculture for monitoring biotic stress in plants due to pathogens and pests. *Inventions* **6**, 29 (2021).

[7] Ali, M. M., Bachik, N. A., Muhadi, N., Yusof, T. N. T. & Gomes, C. Non-destructive techniques of detecting plant diseases: A review. *Physiol. Mol. Plant Pathol.* **108**, 101426 (2019).

[8] Dwivedi, R., Dey, S., Chakraborty, C. & Tiwari, S. Grape disease detection network based on multi-task learning and attention features. *IEEE Sensors J.* **21**, 17573–17580. 10.1109/JSEN.2021.3064060 (2021).

[9] Kianat, J. et al. A joint framework of feature reduction and robust feature selection for cucumber leaf diseases recognition. *Optik* **240**, 166566. 10.1016/j.ijleo.2021.166566 (2021).

[10] Zhao, Y., Sun, C., Xu, X. & Chen, J. Ric-net: A plant disease classification model based on the fusion of inception and residual structure and embedded attention mechanism. *Comput. Electron. Agric.* **193**, 106644. 10.1016/j.compag.2021.106644 (2022).

[11] Shwetha, V., Bhagwat, A. & Laxmi, V. Leafspotnet: A deep learning framework for detecting leaf spot disease in jasmine plants. *Artif. Intell. Agric.*10.1016/j.aiia.2024.02.002 (2024).

[12] Sharma, V., Tripathi, A. K. & Mittal, H. Dlmc-net: Deeper lightweight multi-class classification model for plant leaf disease detection. *Ecol. Informatics* **75**, 102025. 10.1016/j.ecoinf.2023.102025 (2023).

[13] Ali, A. H., Youssef, A., Abdelal, M. & Raja, M. A. An ensemble of deep learning architectures for accurate plant disease classification. *Ecol. Informatics* **81**, 102618 (2024).

[14] Tian, Y., Zhao, C., Lu, S. & Guo, X. Multiple classifier combination for recognition of wheat leaf diseases. *Intell. Autom.& Soft Comput.* **17**, 519–529 (2011).

[15] Rumpf, T. et al. Early detection and classification of plant diseases with support vector machines based on hyperspectral reflectance. *Comput. Electron. Agric.* **74**, 91–99. 10.1016/j.compag.2010.06.009 (2010).

[16] Sheelavantamath, B. Plant disease detection and its solution using image classification. *Int. J. Futur. Res. Dev.* **1**, 73–79 (2020).

[17] Elangovan, K. & Nalini, S. Plant disease classification using image segmentation and svm techniques. *Int. J. Comput.Intell. Res.* **13**, 1821–1828 (2017).

[18] Majumdar, D., Kole, D. K., Chakraborty, A. & Majumder, D. D. An integrated digital image analysis system for detection, recognition and diagnosis of disease in wheat leaves. In *Proceedings of the Third International Symposium on Women in Computing and Informatics*, 400–405 (2015).

[19] Sujatha, R., Chatterjee, J. M., Jhanjhi, N. & Brohi, S. N. Performance of deep learning vs machine learning in plant leaf disease detection. *Microprocess. Microsystems* **80**, 103615. 10.1016/j.micpro.2020.103615 (2021).

[20] El Akhal, H., Yahya, A. B., Moussa, N. & El Alaoui, A. E. B. A novel approach for image-based olive leaf diseases classification using a deep hybrid model. *Ecol. Informatics* **77**, 102276 (2023).

[21] Wu, J. et al. Strawberry disease detection through an advanced squeeze-and-excitation deep learning model. *IEEE Trans. AgriFood Electron.* 1–9, 10.1109/TAFE.2024.3412285 (2024).

[22] Bao, W., Yang, X., Liang, D., Hu, G. & Yang, X. Lightweight convolutional neural network model for field wheat ear disease identification. *Comput. Electron. Agric.* **189**, 106367 (2021).

[23] Ferentinos, K. P. Deep learning models for plant disease detection and diagnosis. *Comput. Electron. Agric.* **145**, 311–318 (2018).

[24] Liang, Q. et al. Pd2se-net: Computer-assisted plant disease diagnosis and severity estimation network. *Comput. Electron. Agric.* **157**, 518–529 (2019).

[25] Bao, W. et al. Identification of wheat leaf diseases and their severity based on elliptical-maximum margin criterion metric learning. *Sustain. Comput. Informatics Syst.* **30**, 100526 (2021).

[26] Jin, X., Jie, L., Wang, S., Qi, H. J. & Li, S. W. Classifying wheat hyperspectral pixels of healthy heads and fusarium head blight disease using a deep neural network in the wild field. *Remote. Sens.* **10**, 395 (2018).

[27] Su, T., Min, S., Shi, A., Cao, Z. & Dong, M. A cnn-lsvm model for imbalanced images identification of wheat leaf. *NeuralNetw. World* **29**, 345–361 (2019).

[28] Saleem, M. H., Potgieter, J. & Arif, K. M. Plant disease detection and classification by deep learning. *Plants* **8**, 468 (2019). 31683734 10.3390/plants8110468 PMC6918394

[29] Kamal, K., Yin, Z., Wu, M. & Wu, Z. Depthwise separable convolution architectures for plant disease classification. *Comput. Electron. Agric.* **165**, 104948 (2019).

[30] Ashwinkumar, S., Rajagopal, S., Manimaran, V. & Jegajothi, B. Automated plant leaf disease detection and classification using optimal mobilenet based convolutional neural networks. *Mater. Today: Proc.* **51**, 480–487. 10.1016/j.matpr.2021.05.584 (2022) (**CMAE &apos;21**).

[31] Gavai, N. R., Jakhade, Y. A., Tribhuvan, S. A. & Bhattad, R. Mobilenets for flower classification using tensorflow. In *2017 international conference on big data, IoT and data science (BID)* 154–158 (IEEE, 2017).

[32] Senthil Pandi, S., Pounambal, M., Jagadeesh, G. & Arivuselvan, K. Advancing agricultural sustainability with gradient weighted densenet-201 model for accurate detection of plant leaf diseases. *Environ. Dev. Sustain.*10.1007/s10668-025-06314-0 (2025).

[33] Senthil Pandi, S., Reshmy, A. K., Muruganandam, S. & Manju, I. Hybrid crossover oppositional firefly optimization for enhanced deep transfer learning in plant leaf disease classification. *J. Crop. Heal.* **77**, 10.1007/s10343-025-01205-w (2025).

[34] Rameshkumar, R., Arunkumar, G., Aruna Devi, B. & Senthil Pandi, S. Enhanced plant disease classification via wild horse optimizer and convolutional-attention bidirectional lstm. *Signal, Image Video Process.*10.1007/s11760-025-04556-z (2025).

[35] Sellam, V., Kannan, N., Senthil Pandi, S. & Manju, I. Enhancing sustainable agriculture using attention convolutional bidirectional gated recurrent based modified leaf in wind algorithm: Integrating ai and iot for efficient farming. *Sustain.Comput. Informatics Syst.* **47**, 101160. 10.1016/j.suscom.2025.101160 (2025).

[36] Murugavalli, S. & Gopi, R. Plant leaf disease detection using vision transformers for precision agriculture. *Sci. Rep.* **15**, 22361 (2025). 40594191 10.1038/s41598-025-05102-0 PMC12216567

[37] Karthik, R., Ajay, A., Bisht, A. S., Illakiya, T. & Suganthi, K. A deep learning approach for crop disease and pest classification using swin transformer and dual-attention multi-scale fusion network. *IEEE Access* (2024).

[38] Arsenovic, M., Karanovic, M., Sladojevic, S., Anderla, A. & Stefanovic, D. Solving current limitations of deep learning based approaches for plant disease detection. *Symmetry* **11**, 10.3390/sym11070939 (2019).

[39] Mohameth, F., Bingcai, C. & Sada, K. A. Plant disease detection with deep learning and feature extraction using plant village. *J. Comput. Commun.* **8**, 10–22 (2020).

[40] Chen, J., Chen, J., Zhang, D., Sun, Y. & Nanehkaran, Y. A. Using deep transfer learning for image-based plant disease identification. *Comput. Electron. Agric.* **173**, 105393 (2020).

[41] Argüeso, D. et al. Few-shot learning approach for plant disease classification using images taken in the field. *Comput. Electron. Agric.* **175**, 105542 (2020).

[42] Nanehkaran, Y., Zhang, D., Chen, J., Tian, Y. & Al-Nabhan, N. Recognition of plant leaf diseases based on computer vision. *J. Ambient Intell. Humaniz. Comput.* 1–18 (2020).

[43] Khamparia, A. et al. Seasonal crops disease prediction and classification using deep convolutional encoder network. *Circuits, Syst. Signal Process.* **39**, 818–836 (2020).

[44] Zhao, Y., Sun, C., Xu, X. & Chen, J. Ric-net: A plant disease classification model based on the fusion of inception and residual structure and embedded attention mechanism. *Comput. Electron. Agric.* **193**, 106644 (2022).

[45] Turkoglu, M., Yanikoğlu, B. & Hanbay, D. Plantdiseasenet: Convolutional neural network ensemble for plant disease and pest detection. *Signal Image Video Process.* **16**, 301–309 (2022).

[46] Assaduzzaman, M. et al. Xse-tomatonet: An explainable ai based tomato leaf disease classification method using efficientnetb0 with squeeze-and-excitation blocks and multi-scale feature fusion. *MethodsX* **14**, 103159 (2025). 40655435 10.1016/j.mex.2025.103159 PMC12255360

[47] Peyal, H. I., Mondal, M. N. I. & Miraz, S. An efficient explainable ai method combining cnn and svm for corn leaf disease detection and visualization. In *2024 27th International Conference on Computer and Information Technology (ICCIT)*, 2958–2962 (IEEE, 2024).

[48] Rashid, M. R. A. et al. An ensemble learning framework with explainable ai for interpretable leaf disease detection. *Array* **26**, 100386 (2025).

[49] Ajay, A. et al. Leafvisionnet: A deep learning-based approach for the classification of black gram leaf disease using attention-driven and semi-local feature boosted squeezenet. *Smart Agric. Technol.* **12**, 101245. 10.1016/j.atech.2025.101245 (2025).

[50] Senthil Pandi, S., Reshmy, A., Elangovan, D. & Vellingiri, J. Ai-driven environmental monitoring for hydroponic agriculture: Excnn-lfcp approach. *Earth Sci. Inform.* **18**, 73 (2025).

