# Correction: Optimised MobileNet for very lightweight and accurate plant leaf disease detection

**DOI:** 10.1038/s41598-026-59120-7

**Published:** 2026-06-23

**Authors:** Vincent Nnamdi Ugwah, Vahid Abolghasemi

**Affiliations:** 1 School of Computer Science and Electronics Engineering, University of Essex, Colchester, UK; 2 School of Engineering and Engineering Technology, Federal University of Technology Owerri, Owerri, Imo State Nigeria

Correction to: *Scientific Reports* 10.1038/s41598-025-27393-z, published online 12 December 2025

The original version of this Article contained an error in the name of author Vincent Nnamdi Ugwah, which was incorrectly given as Ugwah Vincent Nnamdi.

The original Article has been corrected.

